# Ultrahigh specificity in a network of computationally designed protein-interaction pairs

**DOI:** 10.1038/s41467-018-07722-9

**Published:** 2018-12-11

**Authors:** Ravit Netzer, Dina Listov, Rosalie Lipsh, Orly Dym, Shira Albeck, Orli Knop, Colin Kleanthous, Sarel J. Fleishman

**Affiliations:** 1 Department of Biomolecular Sciences, Weizmann Institute of Science, 7610001 Rehovot, Israel; 2 Structural Proteomics Unit, Weizmann Institute of Science, 7610001 Rehovot, Israel; 3 Department of Biochemistry, University of Oxford, South Parks Road, Oxford, OX1 3QU, UK

## Abstract

Protein networks in all organisms comprise homologous interacting pairs. In these networks, some proteins are specific, interacting with one or a few binding partners, whereas others are multispecific and bind a range of targets. We describe an algorithm that starts from an interacting pair and designs dozens of new pairs with diverse backbone conformations at the binding site as well as new binding orientations and sequences. Applied to a high-affinity bacterial pair, the algorithm results in 18 new ones, with cognate affinities from pico- to micromolar. Three pairs exhibit 3-5 orders of magnitude switch in specificity relative to the wild type, whereas others are multispecific, collectively forming a protein-interaction network. Crystallographic analysis confirms design accuracy, including in new backbones and polar interactions. Preorganized polar interaction networks are responsible for high specificity, thus defining design principles that can be applied to program synthetic cellular interaction networks of desired affinity and specificity.

## Abstract (web summary)

The molecular basis of ultrahigh specificity in protein-protein interactions remains obscure. The authors present a computational method to design atomically accurate new pairs exhibiting >100,000-fold specificity switches, generating a large and complex interaction network.

## Introduction

In the evolution of multicellular organisms, pairs of interacting signaling proteins are duplicated and diversified to generate elaborate interaction networks. An illuminating example of expansion through evolution is seen in the fibroblast growth factor (FGF) family and their receptors (FGFRs)^1^, which in humans include 18 homologous FGFs and seven homologous FGFRs^2^. In this network, some ligands are highly specific and effectively bind and activate just one receptor, whereas others are multispecific and bind at least four receptors. This hierarchical network architecture, allowing both insulated signaling through specifically interacting pairs and simultaneous and parallel signaling through multispecific interactions, underlies the many different roles of FGFs in development and physiology. To date, however, the ability to computationally design artificial networks of such complexity has not been demonstrated. Protein-network engineering has therefore relied on fusion to natural protein interaction modules^3–6^ that may exhibit suboptimal stability, affinity, or undesired cross-reactivity with other cellular components. Design of protein-interaction networks is therefore a challenge of both fundamental and practical importance.

The present work focuses on bacterial colicin endonuclease (colE)/immunity (Im) pairs as a model system^7^. It demonstrates how pairs with hugely varying affinities and specificities can be designed from as single complex, collectively forming a protein-interaction network. The colE proteins are nonspecific DNases, which are produced by *Escherichia coli* to eliminate neighboring bacteria. To avoid autotoxicity, the producing cells co-express Im proteins, which tightly bind and inhibit colE’s activity^8^. In the colE/Im system, comprising four homologous pairs (colE^wt2^/Im^wt2^, colE^wt7^/Im^wt7^, colE^wt8^/Im^wt8^, and colE^wt9^/Im^wt9^), each cognate pair forms an ultrahigh affinity complex (*K*_D_ < 10^−^^13^ M), whereas the non-cognate pairs show non-protective affinities (*K*_D_ > 10^−10 ^M)^9,10^. Due to their ultrahigh pairwise specificity (4–10 orders of magnitude specificity switch among homologs), the colicins have served as models for computational specificity design. Previous studies mutated interfacial amino acids and changed the rigid-body orientation of the colE^wt7^/Im^wt7^ pair to block binding to the wild-type partners^11,12^. These and other computational specificity-design studies yielded at most two orders of magnitude difference in affinity between the newly designed partners and undesired interactions between the designed and wild-type proteins^11–19^.

The much larger specificity switches observed in natural systems^2,10,20^ compared to previous computational design studies suggest that sequence and rigid-body changes alone are insufficient to effect large changes in protein-interaction specificity. Indeed, previous structural analyses highlighted the role of backbone changes, including amino acid insertions and deletions (indels) in loop regions, in determining interaction specificity^2,21–23^. Moreover, backbone changes at an interface add many options for encoding alternative interactions among pairs and may facilitate the construction of a diverse network from a single starting pair. The design of new loops and indels, however, is a major unmet challenge in computational protein design due to the many conformational degrees of freedom that the protein backbone can adopt, and in all design of new folds, loops have been too short to support active sites^24–28^. Backbone design at binding sites is further complicated by the requirement to balance protein stability, affinity, and specificity, which can be mutually exclusive outcomes of the design process^22^. Thus, although success was demonstrated in grafting natural binding epitopes from one protein to another^29–34^ and designing loop backbones that lack molecular activity^35,36^, accurate design of loop backbones that encode new interactions at binding sites has remained elusive.

To solve the problem of designing multiple new high-specificity pairs, we first develop a method for binding-site backbone design. Previous specificity-switch methods relied on “explicit” negative design; that is, designing sequence features, such as steric overlaps, to explicitly block pairing with undesired partners, which therefore required atomic structures^11–13,37^. In the design of multiple new high-specificity pairs, by contrast, experimental structures of the designed pairs are not yet available and therefore cannot be used to explicitly design against undesired non-cognate interactions. We hypothesized, however, that design of preorganized, substantially different backbone conformations at the binding interface would be sufficient for encoding binding incompatibility among pairs. This strategy is known as “heuristic” negative design, as it encodes general features (in this case, rigid and different backbones) that destabilize undesired bound states rather than explicitly countering these states^24^. Our results demonstrate that the design of substantially different backbone conformations, orientations, and sequences at the binding site generates dozens of new interaction pairs from a single starting one. Heuristic negative design does not strictly guarantee that all resulting pairs exhibit high specificity. Indeed, the designed pairs collectively form an interaction network comprising ultrahigh specificity binders, four of which exhibit 1000-fold to >100,000-fold pairwise specificity switches relative to the wild-type proteins or other designs, whereas other designs are multispecific and interact with other partners with nearly equal affinity. By contrasting ultrahigh specificity binders and multispecific ones we infer molecular principles which code for specificity and multispecificity.

## Results

### A method for binding-site backbone design

**Fig. 1:**
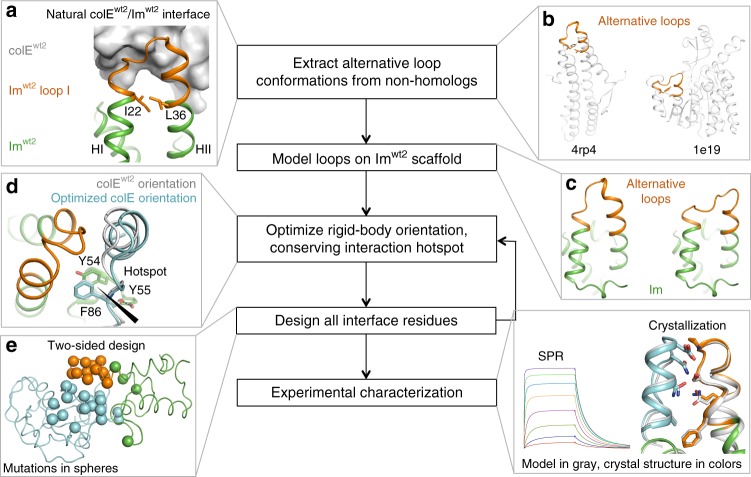
Key steps in the design of high-specificity protein pairs. The algorithm was implemented on the bacterial colicin colE^wt2^/Im^wt2^ complex. **a** Loop I (gold) on Im^wt2^ at the interaction interface was chosen as a site for backbone design. The flanking helices I and II are denoted as HI and HII. The loop stem side chains are shown in sticks. **b** Alternative loops (gold) of diverse conformations and sequence lengths, which exhibit stem geometry compatible to that of loop I, were found in non-homologous protein structures. **c** The matching conformations were placed on the Im instead of the natural loop I. **d** Each Im conformation was docked against the colE using the natural hotspot interaction (in sticks) as a pivot for rotation (indicated in black). **e** For each design pair, the sequence of the entire interface, including loop I and both chains but excluding the hotspot region, was designed (spheres) to maximize colE/Im interaction affinity

Similar to FGF/FGFR and many other protein–protein interactions^2,38^, the molecular structure of the colE^wt2^/Im^wt2^ interface (Protein Data Bank [PDB] entry 3U43) comprises a conserved core, known as the interaction hotspot (comprising Tyr54 and Tyr55 on Im^wt2^ and Phe86 on colE^wt2^), which encodes much of the binding affinity, and peripheral interactions, where binding incompatibility toward other natural colicins is encoded^9,39,40^. Additionally, rigid-body orientation and backbone-conformational differences in loop I, which connects helix I and helix II of the Im protein and is at the periphery of the binding interface, make important contributions to specificity^23,41–43^ (Supplementary Fig. 1). Inspired by this modularity of binding interfaces, we reasoned that specificity design should focus on designing new backbones, including indels, in loop I, while conserving the interaction hotspot and optimizing the rigid-body orientation and sequence of other interfacial regions for the new conformation. Structural analysis identified a pair of geometrically conserved and spatially proximal positions on Im helices I and II that form a stem for loop I (Ile22 and Leu36; Fig. 1a). Since loop I is at the periphery of the binding interface, we hypothesized that backbone designs that retained the stem geometry would allow the remainder of the Im protein, including the essential hotspot, to fold to the native conformation, thereby maintaining high-affinity binding.

Recently, we described a backbone-design method, called AbDesign^44^, and demonstrated that it could assemble backbone fragments from a homologous protein family and design the amino acid sequence to yield functional and atomically accurate antibodies^45^ and enzymes^46^. AbDesign reiles on high structural diversity in the homologous protein family, however, and the limited diversity in colicin immunity proteins (only four molecular structures are available) precluded its effective application in this case. We therefore extended AbDesign to incorporate backbone fragments from non-homologous proteins, searching the PDB for alternative loop backbones that were geometrically compatible with the loop I stem. We identified 2776 segments of 9–21 amino acids (compared to 15 amino acids for loop I of Im^wt2^) that originated in proteins of unrelated folds and functions (Fig. 1b). For each matching segment from the PDB, we used AbDesign to exchange loop I from Im^wt2^ with the matched backbone^44^, resulting in 2657 designed low-energy Im backbones (Fig. 1c). We then relaxed the designed complexes by rigid-body docking. Each docking step rotated the colE/Im design around the hotspot, which served as a pivot^12,42^, conserving the essential hotspot interactions and opening new design opportunities due to the displacement of loop I (Fig. 1d). Furthermore, docking moves were interspersed with sequence-design steps applied to the entire binding surfaces of both the colE and the Im proteins (two-sided design), including loop I (Fig. 1e).

**Fig. 2:**
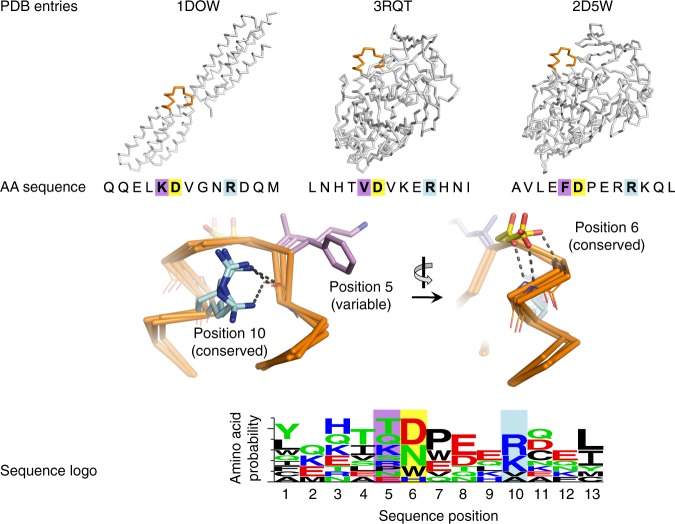
A representative example demonstrating that conformation-specific PSSMs preserve sequence features that preorganize the loop backbone. (Top) Similar loop conformations (orange) arise in non-homologous proteins and are compatible with the Im loop I stem. (Center) Positions on the loops that form stabilizing side chain-backbone interactions are conserved: for instance, positions 6 and 10 (yellow and cyan, respectively). By contrast, the surface-exposed position 5 (purple) is hypervariable. (bottom). Sequence conservation patterns can be used to restrict design choices to those that stabilize the loop conformation, while at the same time, enable large diversity at positions that may interact with the designed partner protein

Sequence design, however, was not allowed to sample all amino acid choices. Rather, Position-Specific Scoring Matrices (PSSMs) were constructed from multiple-sequence alignments of colE and Im homologs of >50% sequence identity, and at each position, only mutations to evolutionarily frequent identities (PSSM scores ≥ 0) were allowed^44,45,47^. The designed loop I segments, however, were not homologous to Im proteins, and PSSMs were therefore built from alignments of non-homologous sequences from the PDB with a similar backbone conformation. Although the multiple-sequence alignments comprised segments extracted from non-homologous proteins of different folds and molecular functions, we noted that the resulting loop I PSSMs were conserved at positions that were likely responsible for preorganizing the loop backbone, for instance, through hydrogen bonds; conversely, the solvent-exposed positions were variable (Fig. 2). We therefore surmised that the sequence-conservation patterns, which arose through convergent evolution of similar backbone conformations evolving in different structural and functional contexts, provided important design constraints for loop backbone preorganization. Solvent-exposed amino acids, by contrast, exhibited high diversity and could be designed to encode new interactions with the designed colE.

The design algorithm thereby searches sequence-conformation space for solutions that balance the demands of binder design, including affinity, specificity, and protein stability^22^. The result of applying this algorithm was 636 designed colE/Im pairs with computed energy and structure characteristics, such as binding energy and interface shape complementarity, that were on par with the natural colicins.

### Ultrahigh specificity among designed pairs

**Fig. 3:**
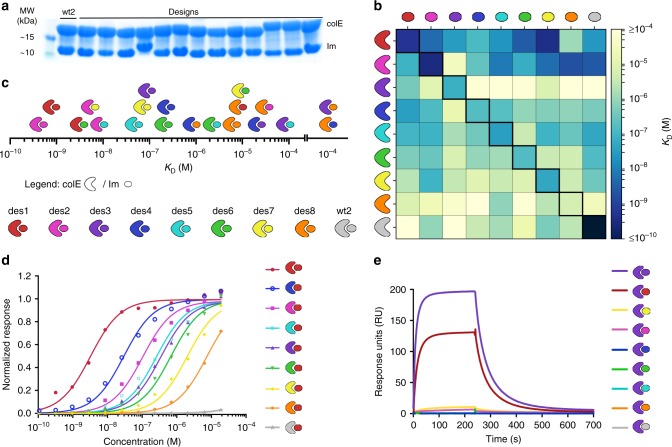
Affinity and specificity in the designed interaction network. **a** Co-expression and affinity-purification of 13 representative colE/Im designs and colE^wt2^/Im^wt2^ indicate that the design pairs express well and interact. **b** Interaction affinities of eight selected designs to their cognate (diagonal) and non-cognate (off-diagonal) partners and to wt2, determined by surface plasmon resonance (SPR). Design color-coding shown to the left of the panel. The colE^wt2^/Im^wt2^ interaction was also measured (Supplementary Fig. 3), but the affinity value reported here (*K*_D_ = 4 × 10^−15 ^ M) was taken from previous stopped-flow measurements, which provide better resolution at ultrahigh affinities^9^. Numerical *K*_D_ values and fitting methods are available in Supplementary Table 1. Kinetic constants and fitted sensograms for the six cognate interactions that were determined kinetically and for colE^wt2^/Im^wt2^ are shown in Supplementary Figure 3. **c** Interaction affinities span seven orders of magnitude, from high picomolar to high micromolar. **d** Im^des1^ shows 59 to 170,000-fold pairwise specificity for its cognate colE^des1^ (red curve) over non-cognate counterparts. Binding of Im^des1^ to cognate and non-cognate colE proteins was measured using SPR at different Im concentrations and normalized to the maximal response at saturating concentrations (*R*_max_) extracted by affinity fitting. **e** colE^des3^ shows 6- to 1400-fold specificity for its cognate Im^des3^ (purple sensogram) over non-cognate partners. SPR binding sensograms are presented at 233 nM Im concentration

Following visual inspection, we selected 59 diverse, low-energy colE/Im pairs that exhibited favorable interfacial interactions for experimental testing (Supplementary Data 1). Each gene encoding a designed colE/Im pair was cloned in tandem into the pET21d expression vector, with the colE gene followed by the Im gene with an intervening two base-pair frameshift^41^. The plasmids were then cloned into T7 Express *lysY/I*^*q*^ *E. coli* strain (NEB), which tightly regulates protein expression. The colE proteins are potent bacteriocins, and clonal sequencing revealed that even under non-inducing conditions, 41 designed colE proteins accumulated spontaneous inactivating mutations. Sequences of the remaining 18 pairs matched the designs, suggesting that in these 18 pairs, the Im proteins either blocked the activity of their colE targets as designed, or that the colE designs were inactive. To resolve this uncertainty, the plasmids containing the colE/Im pairs were transformed into the overexpression *E. coli* strain BL21 and no viable colonies were observed, indicating that the colE proteins were active and toxic in vivo. We next introduced an inactivating point mutation at the colE DNase active site in all designs (His127Ala)^48^ and overexpressed and purified each of the 18 pairs, observing that each colE protein copurified with its Im partner at roughly stoichiometric concentration (Fig. 3a). We therefore concluded that these 18 designed colE/Im pairs indeed formed the cognate interactions in vitro, and that the Im proteins were protective against endonuclease activity in vivo but had lower affinities than cognate wild-type colE/Im complexes.

We next characterized the binding affinity of eight designed pairs that represented high diversity in loop I conformations, including indels. In fact, among these eight, the designed loop I backbones and sequences were more different from one another than those of the wild-type Im2 and 9 (Supplementary Fig. 2). The designed pairs and the parental pair colE^wt2^/Im^wt2^ were expressed (with the inactivating His127Ala mutation), purified, and subjected to all-against-all binding analysis using surface-plasmon resonance (SPR). The resulting 9 × 9 protein-interaction matrix revealed that the designed-cognate affinities spanned four orders of magnitude, from high picomolar to low micromolar. The non-cognate interactions further extended the affinity range to high micromolar affinities (Fig. 3b; Supplementary Table 1, Supplementary Figs. 3 and 4) (see Methods for details on SPR analysis). Viewed as a network of interacting proteins (including all 8 × 8 cognate and non-cognate interactions), the designed pairs spanned at least seven orders of magnitude in affinity (Fig. 3c), covering the entire range of non-obligatory interactions observed in biology at the physiological concentrations of cellular proteins.

**Fig. 4:**
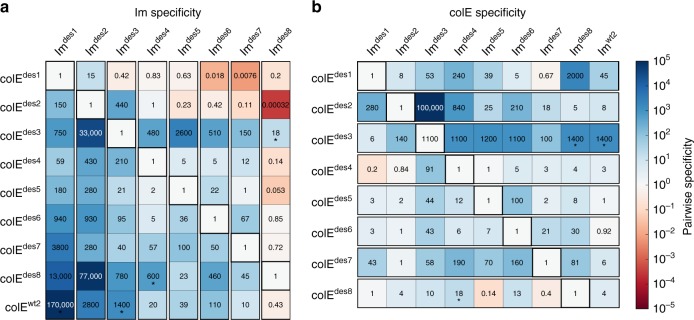
Ultrahigh specificity and multispecificity among the designed pairs. Pairwise specificity values represent the fold-change in affinity of **a** the Im and **b** colE proteins against each non-cognate counterpart relative to the cognate affinity (assigned a value of 1) according to Eq. (1). Values marked with an asterisk represent lower bounds of pairwise specificity, as the experimentally measured interaction affinity exceeded 10^5^ nM

The affinity matrix revealed large differences in pairwise specificity, that is the ratio between the non-cognate and the cognate binding affinities. For instance, the cognate-designed colE^des1^/Im^des1^ exhibited high binding affinity (*K*_D_ = 0.58 nM), whereas the affinity of Im^des1^ for other colE proteins was at least 59-fold and up to 170,000-fold weaker (>5 orders of magnitude pairwise specificity switch) (Fig. 3d). Notably, the affinity of the non-cognate complex colE^wt2^/Im^des1^ was below the SPR detection limit and was estimated at >10^5^ nM. Moreover, design pairs colE^des2^/Im^des2^ and colE^des3^/Im^des3^ showed at least three orders of magnitude pairwise specificity switches relative to either one or both of the colE^wt2^ and Im^wt2^ proteins; they also exhibited such high specificity switches relative to three and four other designs, respectively, confirming that the design algorithm reproducibly generated high-specificity pairs (Figs. 3e and 4 and Supplementary Fig. 5). These pairwise specificity switches exceeded those attained by past design studies by as much as three orders of magnitude^11–13,15^. Nevertheless, not all designed pairs exhibited ultrahigh pairwise specificities. For example, colE^des7^/Im^des7^ exhibited relatively high cognate affinity (51 nM), but Im^des7^ bound three non-cognate colE proteins with similar or up to two orders of magnitude higher affinities (Fig. 4). Thus, collectively the designed pairs yielded a complex interaction network comprising both ultrahigh specificity interactions and multispecific binders.

To quantify the network specificity for each designed Im and colE protein, we computed the parameter *α*, which expresses the steady-state fraction of protein that binds cognate-designed ligand relative to the non-cognate ligands when all ligands compete for binding the protein at a constant predefined concentration^49^ (Supplementary Table 2). The highly specific Im^des1^, for instance, shows an *α*_1nM_ value of 11.8, meaning that at 1 nM concentration, the fraction of Im^des1^ bound to colE^des1^ exceeds by more than an order of magnitude the fraction bound to the other seven designed colE proteins, combined. Im^des6^, Im^des7^, and Im^des8^, by contrast, exhibit *α*_1nM_ values <0.1 and are expected to bind multiple colE proteins at this concentration.

### Optimization of affinity and specificity by design

**Fig. 5:**
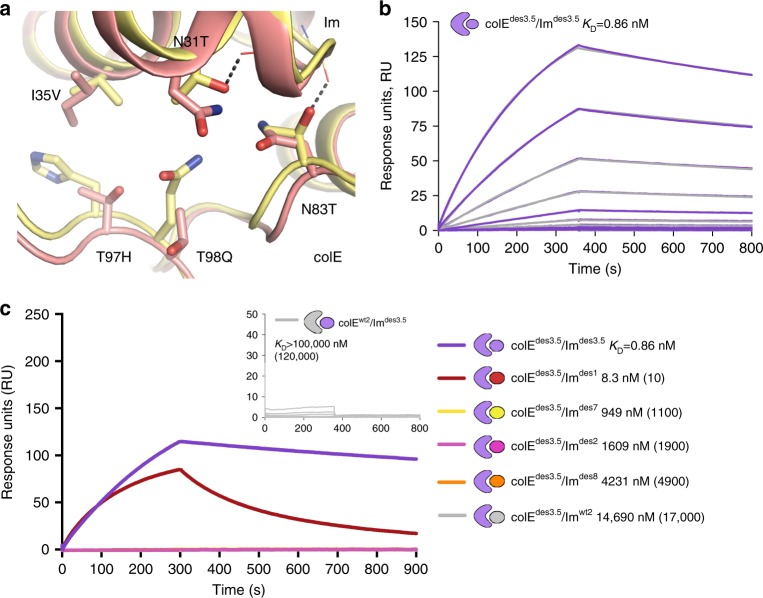
Large gains in affinity and specificity using AffiLib affinity-enhancement design. **a** Low-energy combinations of mutations (sticks) were designed at the colE^des3^/Im^des3^ interface. Model structure of the starting colE^des3^/Im^des3^ pair and model of the higher-affinity colE^des3.5^/Im^des3.5^ are shown in salmon and yellow, respectively. **b** SPR analysis for colE^des3.5^/Im^des3.5^ shows cognate affinity of 0.86 nM (data in light purple; fits in gray), relative to 73 nM for colE^des3^/Im^des3^ (Supplementary Fig. 3). *K*_D_ was determined by fitting to a single-exponential kinetic model (*k*_on_=4.15 × 10^5^  M^−1^ s^−^^1^, *k*_off_=3.57 × 10^−4^  s^−1^). **c** Improved pairwise specificity in colE^des3.5^/Im^des3.5^ relative to the original design pair. colE^des3.5^ shows 10–10^4^-fold affinity difference between cognate (light purple) and non-cognate interactions (colE^des3^ pairwise specificity values in parentheses). The SPR sensograms were collected at 0.96 nM Im concentrations. Inset: Im^des3.5^ shows no detectable binding to colE^wt2^ in concentrations up to 56.7 μM, translating to a pairwise specificity switch of 10^5^ (in parentheses)

While two designed pairs (colE^des1^/Im^des1^ and colE^des2^/Im^des2^) exhibited subnanomolar affinities, the majority had affinities in the range of 30–200 nanomolar, orders of magnitude weaker than those of natural colicin cognate pairs. To test whether cognate affinity could be improved, we focused on colE^des3^/Im^des3^, which exhibited *K*_D_ = 73 nM and at least three orders of magnitude pairwise specificity relative to colE^wt2^/Im^wt2^ (Fig. 4). Current methods for experimental in vitro evolution of pairs of interacting proteins lack robustness, however, and have not been broadly applied, particularly to cytotoxic proteins such as endonucleases. We therefore developed a computational affinity-design method that introduced mutations simultaneously to both interacting proteins. Briefly, we used Rosetta to model 10^5^ unique colE^des3^/Im^des3^ mutants, each encoding a different combination of 3–7 mutations on both the Im and the colE binding surfaces and ranked them by computed binding energy (details in Methods). Following visual inspection, we selected 19 low-energy mutants that exhibited high diversity relative to one another and cloned these designs into the high-expression *E. coli* strain BL21. One of the designs, colE^des3.5^/Im^des3.5^ with five mutations relative to colE^des3^/Im^des3^ (Fig. 5a) was as viable as the wild-type pair colE^wt2^/Im^wt2^, compared to complete inviability in this bacterial strain for any of the previously designed pairs, suggesting that this design exhibited the highest affinity in the designed set. SPR analysis confirmed that colE^des3.5^/Im^des3.5^ improved affinity by two orders of magnitude (*K*_D_ = 0.86 nM, Fig. 5b) through 52-fold decrease in off-rate and twofold increase in on-rate relative to colE^des3^/Im^des3^ (Supplementary Fig. 3). Furthermore, the affinity of the non-cognate pair colE^wt2^/Im^des3.5^ remained weaker than the SPR detection limit (*K*_D_ > 10^5^ nM), translating to >5 orders of magnitude pairwise specificity switch. The affinity of colE^des3.5^ for non-cognate Im proteins was increased compared to colE^des3^, and yet the pairwise specificity was either improved by an order of magnitude or remained unchanged. For example, the pairwise specificity of colE^des3.5^ against binding Im^des2^ was 1900-fold, compared to 140-fold specificity of colE^des3^ against Im^des2^ (Fig. 5c). Hence, cognate affinities of the designed pairs could be substantially improved through another round of design, while retaining, and even improving, specificity.

Note that this automated design method achieved substantial affinity and specificity enhancement by testing only 19 mutants, and we recently showed that a similar method applied to enzyme active sites can result in orders of magnitude improvement in promiscuous catalytic efficiencies^50^. The affinity enhancement we observed here is comparable to that achieved in past binder design studies only through laborious iterations of random or focused mutagenesis, deep sequencing, and selections^51–53^. To enable wide access to this affinity-enhancement design method, we developed a web server, which we called the Affinity Library or AffiLib (http://AffiLib.weizmann.ac.il). AffiLib starts from the molecular structure of an interacting pair and designs a library of potentially enhanced binders (either both interacting proteins are designed, as exemplified here, or only one of them, depending on the user’s choice). We anticipate that AffiLib may in some cases eliminate the laborious iterations of affinity maturation or deep mutational scanning that are a requirement in most current binder design and engineering studies^54,55^.

### The structural basis of specificity

**Fig. 6:**
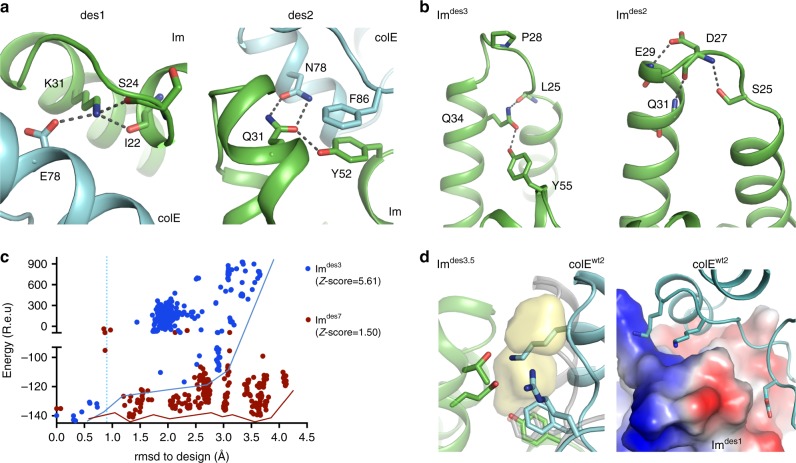
The structural basis of specificity and multispecificity among designed pairs. **a** Structure models show high polarity in designed, high-specificity interfaces. Im^des1^ buries positively charged Lys31 at the interface, which is compensated by negatively charged Glu78 on the cognate colE^des1^ and by hydrogen bonding to backbone atoms on Im loop I. colE^des2^/Im^des2^ interact through a hydrogen-bond network that involves hotspot residue Tyr52. **b**, **c** Im Loop I preorganization predicts Im specificity. **b** Structure models show that stabilizing interactions within loop I of the high-specificity designs preorganize the loop I backbone in the designed conformation. Loop I of Im^des3^ is configured by Pro28 and side chain-backbone hydrogen bonds. Loop I of Im^des2^ is stabilized by a network of side chain-backbone polar interactions. **c** Modeling the sequence of the high-specificity Im^des3^ on alternative backbone conformations observed in the Protein Data Bank converges on low-energy conformations only in the vicinity of the designed backbone conformation (blue; vertical cyan line at 0.9Å rmsd). Models of the multispecific Im^des7^, by contrast, diverge to multiple minima away from the designed conformation (red). *Z*-scores indicated in parentheses. **d** Models of the relaxed non-cognate colE/Im pairs suggest that low binding affinity results from cavities, electrostatic repulsion and unsatisfied polar atoms at the interface. The model of colE^wt2^/Im^des3.5^ (left), with *K*_D_  ≥ 100,000 nM, shows a large cavity (yellow) in loop I region and reorientation of the hotspot Phe86 (cognate colE^des3^ orientation in gray). Model of colE^wt2^/Im^des1^ (right), with *K*_D_ ≥ 100,000 nM, shows positively charged Lys on colE^wt2^ that faces the positively charged loop I region on Im^des1^ (surface colored according to the electrostatic potential), and a negatively charged Glu that faces a surface of the same charge on helix II of Im^des1^. The computed binding energies for these non-cognate interactions are poor (−12.4 Rosetta energy units [R.e.u] for colE^wt2^/Im^des3.5^ and −15.8 R.e.u for colE^wt2^/Im^des1^) compared to tight binding of the cognate colE^des3.5^/Im^des3.5^, colE^des1^/Im^des1^, and colE^wt2^/Im^wt2^ counterparts (−36.0, −39.9, and −34.6 R.e.u, respectively)

The molecular underpinnings of specificity in natural protein-interaction networks are often obscured by evolutionary drift and functional constraints outside the binding interfaces. Among the designed pairs, by contrast, changes were localized to the designed interfaces only, and all designs were >80% sequence identical to the wild type, allowing us to focus attention exclusively on the binding interface (Supplementary Data 1). Visual inspection suggested that among the Im designs that exhibited high network specificity, most had polar binding surfaces. For instance, the highly specific Im^des1^ (*α*_1nM_ = 11.8) bound its cognate colE^des1^ using a buried positive charge (Lys31) that was stabilized by a countercharge (Glu78) on colE^des1^ and by hydrogen bonds; colE^des2^/Im^des2^ (Im^des2^ exhibited network specificity *α*_1nM_=3.7) formed a hydrogen-bonding network within each partner and across the interface, including a hydrogen bond with the Im hotspot residue Tyr52 that is not seen in the parental interaction (Fig. 6a). Since polar contacts are geometrically highly constrained, such interactions may enhance specific molecular recognition. Note that the strategy of burying charged or polar residues that are not compensated by non-cognate partners is also observed in natural ultrahigh-specificity pairs^56^. Nevertheless, design of polar interactions at binding sites was until now only demonstrated in homo oligomeric coiled coils^57^ and was considered a major unmet challenge for binder design^24–26,51^.

The objective of our study was to design new high-specificity pairs. Indeed, both the Im and the colE proteins of design pairs 1–3 showed >3 orders of magnitude pairwise specificity switches relative to at least one of the non-cognate proteins (Fig. 4). Other designs, by contrast, interacted with several non-cognate partners with similar or even higher affinities, allowing us to also examine the molecular basis of high specificity versus multispecificity. We first focused on high-specificity Im designs, since they could reveal relationships between backbone design and binding specificity. Visual inspection suggested that high-specificity Im design models had more preorganized loop I backbones. For instance, in loop I of Im^des3^, Pro28 constrained the conformation space of neighboring amino acids, and in Im^des2^, Ser25 stabilized the backbone by hydrogen bonding to the backbone amide nitrogen of Asp27 (Fig. 6b). To systematically quantify preorganization in backbone design, we used Rosetta to compute a putative conformational landscape for each designed loop I. We threaded each of the designed Im sequences on all of the Im conformations of the same sequence length in our backbone database and computed the energy of the resulting Im models in isolation from their colE partners, thus generating for each sequence a landscape of conformations and their associated energies. For each landscape, we calculated the *Z*-score (see Methods), which reflects how well the designed loop I backbone conformation is energetically discriminated from alternative conformations; a large energy gap between the designed conformation and alternatives (large *Z*-score) predicts that the sequence is more stable in its designed conformation relative to alternatives and hence is more preorganized^58^. The Im proteins, Im^des1^, Im^des2^, and Im^des3^, showed a large energy gap (*Z*-scores 2.6, 3.3, and 5.6, respectively), as was observed for the natural Im^wt2^ (*Z*-score 3.6). By contrast, multispecific designs, such as Im^des6^ and Im^des7^, exhibited low *Z*-scores (1.4 and 1.5, respectively), including, in some cases, alternative backbone conformations of lower energy than the designed conformation (Fig. 6c and Supplementary Fig. 6). These results therefore suggested that preorganized backbone conformations were more likely to result in high-specificity binding.

We next tested whether computational modeling could provide structural insights into the remarkable incompatibility between some of the non-cognate pairs. To allow reliable modeling, we focused on colE/Im pairs that showed high-affinity cognate binding and for which our conformational-landscape analysis suggested that the Im loop I backbone was preorganized (Im^des1^, Im^des2^, Im^des3^, Im^des3.5^, and Im^wt2^). Using Rosetta, we computed models for both cognate and non-cognate pairs. As expected, models of the cognate pairs (*K*_D_ < 80 nM) showed favorable predicted binding energy (<−34 Rosetta energy units [R.e.u]). Interestingly, design pairs 3 and 3.5, which differed by five mutations and exhibited experimentally determined cross-binding affinities similar to those of the cognates (Supplementary table 1) were also predicted to have cognate affinities for their cross-interactions (<−35 R.e.u; Supplementary Fig. 7 and Supplementary Table 3). As expected, Rosetta did not discriminate between non-cognate pairs of high (*K*_D_ < 100 nM) and intermediate affinities (*K*_D_ 100–1000 nM); these pairs generally showed computed binding energies of approximately −27 R.e.u, higher than cognate pairs but lower than the weak non-cognate ones. In contrast, non-cognate pairs with weak affinities (*K*_D_ > 1000 nM) had much higher predicted binding energies (>−27 R.e.u). Furthermore, the interactions of colE^wt2^ with Im^des1^, Im^des3^, and Im^des3.5^ (*K*_D_ ≥100,000 nM) showed extremely unfavorable calculated binding energies (>−16 R.e.u). Visual inspection of these non-cognate models revealed substantial packing defects and in some cases same-charge repulsion and unpaired polar amino acid side chains. Thus, preorganized and incompatible backbone conformations precluded the formation of the hotspot region and peripheral polar networks at the interface of non-cognate colE and Im proteins, providing a possible molecular explanation for the observed low affinities in these non-cognate pairs (Fig. 6d).

**Fig. 7:**
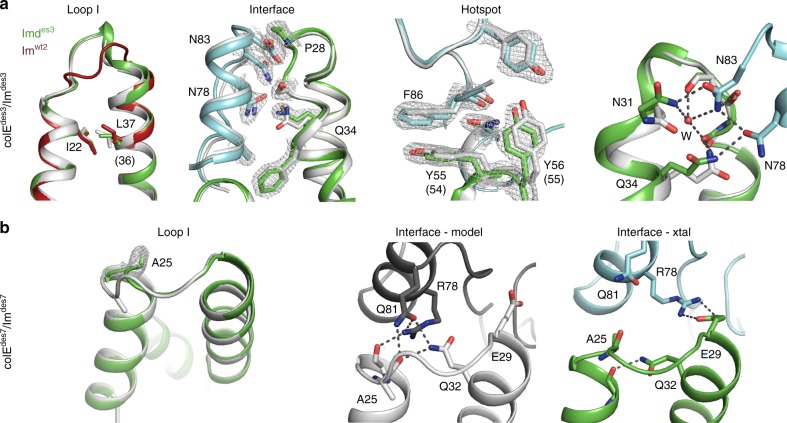
Crystallographic analysis of specific and multispecific colE/Im pairs. **a** The crystal structure of the specific colE^des3^/Im^des3^ (cyan/green, PDB entry 6ERE) verifies the accuracy of the computational design (gray). The designed Im loop I backbone is within 0.5 Å Cα rmsd and the interactions between the colE and Im, including the hotspot residues, are atomically accurate, as is the rigid-body orientation. A water molecule (w), which was not modeled by Rosetta, participates in the designed interfacial hydrogen-bond network. The large difference in loop I conformation and indels relative to Im^wt2^ (16 amino acids in Im^des3^ compared to 15 in Im^wt2^, in scarlet) and the other designs promote specificity. Numbers in parentheses refer to Im^wt2^ numbering. **b** The crystal structure of the multispecific colE^des7^/Im^des7^ (cyan/green, PDB entry 6ER6) shows a local conformational change relative to the design in loop I centered on Ala25, but the backbone conformation is within 0.7 Å Cα rmsd of the design model. This local conformational change prevents the formation of a designed hydrogen-bond network

To verify the atomic accuracy of the design procedure, we determined the structures of two designed pairs by X-ray crystallography: colE^des3^/Im^des3^ with >3 orders of magnitude pairwise specificity relative to the wild-type colE^wt2^/Im^wt2^ and colE^des7^/Im^des7^ with low Im specificity. In both structures, the conserved hotspot region formed as predicted. Furthermore, the high-specificity design, colE^des3^/Im^des3^ showed high accuracy throughout loop I (0.5 Å Cα rmsd over all Im side chains). The designed rigid-body orientation and side-chain conformations at the interface were also atomically accurate, except two polar side chains (Asn31 and Gln34) that reoriented due to a water molecule that was not modeled by Rosetta. Apart from this difference, the polar interaction network in this complex formed with remarkably high accuracy compared to the designed one (Fig. 7a). In the multispecific colE^des7^/Im^des7^, however, we noted a conformational change in loop I localized around Ala25, while the rest of the loop was atomically accurate (0.7 Å Cα rmsd on all Im side chains). Nevertheless, this local difference relative to the design conception prevented the formation of a designed hydrogen-bond network (Fig. 7b). This local conformational change was partly predicted by the conformational-landscape analysis above, according to which multiple low-energy conformations were compatible with the designed sequence (Fig. 6c).

Thus, structural and computational analyses collectively suggest that interface polarity and backbone preorganization underlie ultrahigh pairwise specificity in the designs. Due to backbone preorganization, the designed polar interactions can only form accurately in the cognate pairs as seen in the crystallographic analysis of the high-specificity pair colE^des3^/Im^des3^ (Fig. 7a and Supplementary Table 2). Conversely, in the non-cognate pairs that exhibit preorganized backbones, the presence of polar but unsatisfied groups leads to frustrated binding as seen in the computational docking analysis (Fig. 6d). Finally, backbone flexibility may disrupt the formation of the designed polar interactions, as seen in the crystal structure of colE^des7^/Im^des7^ (Fig. 7b), enabling alternative binding modes and leading to multispecific molecular recognition as for Im^des7^ (Fig. 6c and Supplementary Fig. 5). We therefore conclude that “heuristic” negative design^24^, in which each designed pair is optimized individually for rigid and substantially different backbone conformation than the other pairs, can result in ultrahigh specificity pairs without explicitly designing incompatible interactions among them.

### Interaction networks exhibiting diverse specificity patterns

**Fig. 8:**
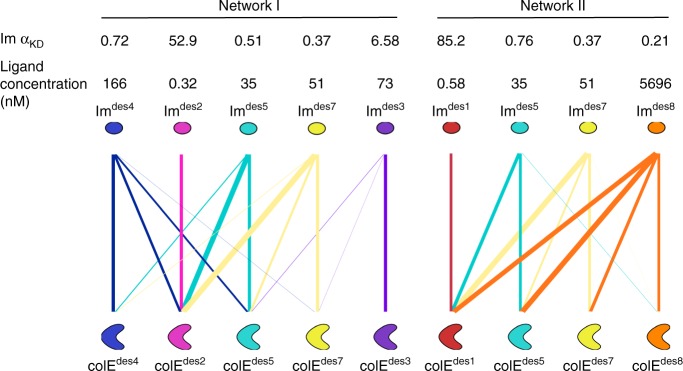
Diverse interaction networks among the designed pairs. The network specificity of each Im for its cognate colE (α_*K*D_; Eq. (3)) is noted on top. The width of the line that connects each colE/Im pair is proportional to the steady-state fractional occupancy of the bound state of this colE by the Im (Eq. (2)). Interactions with fractional occupancy <1% are not shown. Network I: a complex network architecture exhibiting both high specificity (Im^des2^) and multispecificity (Im^des7^). Network II: Im^des1^ binds only its cognate colE; Im^des5^ binds two colE proteins; Im^des7^ binds three; and Im^des8^ binds to all colE proteins in the network, thereby exhibiting a hierarchical, “Russian-doll” like network architecture. Scripts for generating interaction networks between selected sets of designed pairs are provided in Supplementary Data 2

The designs can be viewed as a protein–protein interaction network comprising 8 × 8 high-homology pairs. We plotted the expected steady-state binding patterns for two subnetworks, one 5 × 5 and another 4 × 4 (Fig. 8). The first network revealed an architecture similar to some of the most complex signaling networks in humans, such as the FGF/FGFR network^2^. In both the natural and computed networks, some proteins selectively bound one or two partners, whereas others bound multiple partners. The computed network, however, spanned a wider range of affinities, potentially providing greater room for molecular control. The other subnetwork appeared highly hierarchical, reminiscent of a “Russian-doll” pattern, where one Im selectively bound only one colE, a second bound two, a third bound three, and a fourth bound four. Hundreds of other subnetworks can be constructed from these data at different protein concentrations and compositions (Supplementary Data 2), providing a large resource for the design of binding modules of different affinities and specificities (Fig. 8).

## Discussion

Design of high-specificity interactions must consider multiple molecular objectives, including protein affinity for a variety of molecular targets^22,49^. It furthermore requires an ability to design substantial conformational changes at the binding interface, including indels^21^. We presented an algorithm that uses molecular structures of natural proteins to design new binding-site backbone conformations. Sequence alignments of non-homologous but structurally similar backbone conformations provided constraints that restricted design calculations to form stabilizing side chain-backbone contacts that are essential for backbone preorganization. This procedure resulted in several ultrahigh specificity pairs as well as multispecific ones and therefore in a large and complex network of homologous interaction pairs. We also demonstrated that affinity and specificity could be readily enhanced in the designed pairs by applying the automated, web-accessible AffiLib method. More generally, AffiLib may in some cases eliminate the reliance on tedious experimental affinity maturation in protein design and engineering studies^54,55^.

In many cellular interaction networks, individual binding modules, such as SH2 and SH3, exhibit low specificity, and large specificity switches are realized by tethering multiple binding modules^49,59,60^. In the designed pairs, by contrast, ultrahigh specificity relative to the starting pair did not require tethering multiple domains. We are unaware that a relationship between preorganization and specificity was previously noted in natural cellular interaction networks, but it has been demonstrated, for instance, in antibody–antigen recognition. Specifically, the backbones of germline antibodies are often flexible, enabling low-affinity recognition of multiple antigens by adopting different backbone conformations. During affinity maturation, by contrast, mutations preorganize the backbone and enhance antigen specificity^61,62^. Since our results demonstrate that design of new and preorganized backbones at an interface can lead to multiple new high-specificity interactions, we speculate that a similar mechanism may have been exploited by evolution in at least some natural cellular interaction networks.

Many other challenging protein design problems may gain from our specificity-design approach, including design of orthogonal signaling modules^13,18,37^ and enzyme selectivity switches^46,63^. Indeed, binding specificity and enzyme selectivity switches in natural protein evolution are often accompanied by indels at interface backbone segments, as in our design algorithm, rather than just by surface sequence changes^2,21,64^.

We anticipate that the designed network of colE/Im pairs may also be used to program synthetic interaction networks by serving as protein–protein interaction modules or adaptors that facilitate specific or multispecific interactions, as desired. In nature, proteins involved in the same signaling or metabolic pathway are often tethered to a scaffold protein, increasing pathway productivity^3,65^. The affinity matrix provides a resource, from which one could draw subsets of pairs with desired combinations of low or high affinity, as well as insulated or multispecific binding, to design desired wiring diagrams for synthetic multienzyme pathways. The designs are, to the best of our knowledge, orthogonal to eukaryotic cellular systems, thereby providing a highly controlled system for accurate pathway programming. The rules we defined for generating a large network from a single pair of interacting proteins can be applied, in principle, to any interacting pair of proteins of known structure.

## Methods

### A database of alternative conformations for Im loop I

We implemented an algorithm in RosettaScripts^66^ called SSMotifFinderFilter that searched all high-resolution (≤2.2 Å; 60,422 PDB entries) crystal structures in the PDB for pairs of α helical amino acid positions separated by 9–21 positions on the primary sequence that furthermore superimposed the backbone atoms of the Im^wt2^ loop I stem (Ile22 and Leu36) and the preceding and succeeding positions (six positions in all) within 0.61 Å root mean square deviation (rmsd). Specifically, the rmsd calculation was performed on the backbone heavy atoms (N, Cα, C, O) of six pairs of positions: Im^wt2^ 21–23 and 35–37, and three amino acids at the beginning and the end of each matched loop.

### PSSMs for colE, Im, and Im loop I

For the colE and Im proteins, we generated multiple-sequence alignments (MSAs) that were based either on the four sequences of the natural colicin Im and colE 2, 7, 8, and 9 proteins, or on sequences of >50% identity to colE^wt2^ and Im^wt2^ that were collected using BLASTP^67^ on the non-redundant (nr) database. The PSSMs were generated as described in ref. ^47^. For each of the alternative loop I conformations, we generated a PSSM using PSI-BLAST^68^ with one of the following inputs: (i) sequences that encoded a similar backbone conformation that were identified based on pairwise alignment to the input sequence of the backbone heavy atoms (N, Cα, C, O) with rmsd <2 Å for each amino acid. The pairwise alignment algorithm (RotLibOutMover) is implemented in RosettaScripts. The resulting sequences were clustered using cd-hit^69^, with a clustering threshold of 90% sequence identity and default parameters. (ii) For singleton conformations (no conformational homologs with <2 Å pairwise rmsd) we used the BLOSUM62 scoring matrix as a PSSM^70^.

### Im loop I backbone exchange

The structure of E^wt2^/Im^wt2^ (PDB entry 3U43) was minimized in Rosetta using the protocol described in ref. ^47^, and the structures of the monomers were separated. We used AbDesign^44^ to exchange Im^wt2^ loop I (amino acids Ile22 to Leu36) with each of the backbone conformations in the database, and the structure was relaxed using cyclic-coordinate descent (CCD). During this process, conservative mutations (sequence design) were allowed to accommodate loop I to the context of Im^wt2^ with PSSM score ≥2 at each position. The Im stem region is compatible with both the Im backbone and the backbone derived from the conformation database, and accordingly we encoded three different options for the sequence space of allowed mutations on the Im stem: (1) based on the PSSM of Im proteins; (2) based on the PSSM generated for the alternative loop conformation; and (3) a hybrid, in which the Nʹ stem region was based on the Im PSSM and the Cʹ stem region was based on the PSSM of the alternative loop conformation. For each designed Im backbone, all three options of stem PSSM were used for loop I backbone exchange, and the Im design with the lowest energy among the three was selected. In total, 2657 of the conformations in the database were successfully placed on Im2 instead of the wild-type loop I, with backbone heavy-atom rmsd<0.5 Å between each loop conformation in its natural protein context and after placement.

### colE/Im interface design

During design, we used two versions of the Rosetta energy function: the all-atom energy function (talaris2014)^71^ which is dominated by van der Waals, implicit solvation, Coulomb electrostatics, and hydrogen bonding, and a soft-repulsive energy function, in which the van der Waals overlaps and residue conformational strain are attenuated. The energy functions were modified to favor amino acids with higher PSSM scores and with harmonic restraints on the Cα coordinates of the Im to prevent large backbone movements during minimization. In the first step, the structure of colE^wt2^ was added to the model of each Im conformation using the rigid-body orientation of colE^wt2^/Im^wt2^. Then, a new orientation was sampled randomly by rotating the colE around the Im by up to 10^o^ around a pivot that connects the amide nitrogen of hotspot residue Tyr55 on the Im and the Cα of Ala87 on colE. This step preserved the hotspot interaction and generated orientations that were different from the wild-type structure. Next, the sequences of both colE and Im at the interface (up to 10 Å) were designed to optimize binding energy using the soft-repulsive energy function, followed by all-atom docking with soft-repulsive energy. Last, sequence-design and model refinement were performed through four iterations of mutations (except in hotspot residues), side-chain packing and backbone, side chain, and rigid-body minimization using the talaris2014 hard-repulsive energy function. The allowed amino acids for design at each position on the colE and Im were those with PSSM score ≥0. For each of the starting 2657 Im models, the design protocol was applied 100 times.

### Design evaluation

For each of the starting 2657 Im conformations, the structure with lowest colE/Im binding energy was selected among the 100 design trajectories. A set of filters was then applied to select designs with favorable energies and structural characteristics (values for colE^wt2^/Im^wt2^ in parentheses): Im stability<−140 R.e.u (−148 R.e.u); colE stability<−215 R.e.u (−242 R.e.u), colE/Im binding energy<−32 R.e.u (−40.5 R.e.u), colE/Im interface shape complementarity (*Sc*)^72^>0.64 (0.66), colE/Im packing statistics (Packstat)^73^>0.69 (0.694) and solvent accessible surface area buried upon complex formation (SASA) >1600 Å^2^ (1723 Å^2^). In total, 636 colE/Im designs passed these filters and were sorted by their binding energy.

### Pairwise specificity

The specificity of an Im or colE protein for its cognate counterpart relative to another non-cognate one is defined as the ratio between the dissociation constants of the non-cognate interaction and the cognate one, as given by the equation

1 $$\frac{{K_{\mathrm D},{\mathrm {non}}-{\mathrm {cognate}}}}{{K_{\mathrm D},{\mathrm {cognate}}}}.$$

### Network specificity parameter (*ɑ*)

We define the fractional occupancy (*f*) as the fraction of Im (or colE) bound to a colE (or Im) ligand at a predefined ligand concentration, as given by the equation

2 $$f = \frac{1}{{1 + \frac{{K_{\mathrm D}}}{{[L]}}}},$$

where *K*_D_ is the experimentally determined *K*_D_ of this particular colE/Im interaction and [*L*] is the ligand concentration. The network specificity parameter *α* is then defined as the fraction of Im (or colE) bound to its cognate colE (or Im) ligand (*f*_1nM,cognate_) relative to all non-cognate ligands (*f*_1nM,non-cognate_) at a chosen concentration, for example 1 nM

3 $$\alpha _{1\,{\mathrm {nM}}} = \frac{{f_{1\,{\mathrm {nM}}},{\mathrm {cognate}}}}{{\mathop {\sum }\nolimits_{{\mathrm {noncognates}}} f_{1\, {\mathrm {nM}}},{\mathrm {noncognate}}}} \cdot$$

These equations hold under the assumption that the Im (or colE) concentration is much lower than ligand concentration, which therefore remains constant^49^.

### Computational affinity-design

Visual inspection identified seven positions on the colE^des3^/Im^des3^ interface that formed close contacts (Asn83, Thr97, Thr98 on colE^des3^ and Asn31, Gln34, Ile35, Val38 on Im^des3^). We defined the “tolerated sequence space” at these positions as all identities that had PSSM scores ≥−1 and for which Rosetta ΔΔ*G*_bind_ calculations for each individual mutation were at most mildly destabilizing (<2 R.e.u). We next enumerated all possible combinations of mutations within the tolerated sequence space that differed from the starting pair by at least three mutations (103,752 sequences), modeled them in Rosetta and relaxed the models by side-chain packing and backbone, side chain, and rigid-body minimization with harmonic restraints on the Cα coordinates. Many of the top-ranking designs were very similar to one another in sequence. We therefore chose variants that differed from one another by at least three mutations, ranked them by binding energy, and selected 19 variants from the top 50. A web-accessible version of the algorithm is available in the AffiLib web server (http://AffiLib.weizmann.ac.il), which provides user control over amino acid positions for design, the allowed sequence space at each position, and whether to design one or more of the interacting proteins. The web server uses the more recent Rosetta energy function ref2015^74^ and allows selection of different ΔΔ*G*_bind_ and PSSM cutoffs when computing the tolerated sequence space for design. The MSA and PSSM are generated automatically for the entire protein sequence (see ref. ^50^ for details), and are based on sequence homologs above a certain identity threshold, which can be set by the user.

### Conformational-landscape analysis

The sequence of each query Im design was modeled on the backbone conformation of all Im proteins in the database with the same loop I length as the query. The models were relaxed by four iterations of side chain packing and backbone and side-chain minimization using the talaris2014 energy function, and the resulting models’ energies were plotted against the root mean square deviation (rmsd) of their backbone heavy atoms relative to the query Im design. Conformations with rmsd<0.9 Å from the query were defined as near-native. For each resulting conformational landscape, we defined

4 $${Z{\mathrm -}{\mathrm {score}}} = \frac{{\mu _{{\mathrm {nonnative}}} - {\mathrm {min}}_{{\mathrm {near}}-{\mathrm {native}}}}}{{\sigma _{{\mathrm {nonnative}}}}},$$

where *μ*_nonnative_ is the average on all non-native energies; min_near-native_ is the minimum-energy conformation within the native set; and *σ*_nonnative_ is the standard-deviation of the non-native conformations’ energies.

### Structural and energetic analysis of non-cognate complexes

For each Im protein with *Z*-score >2.5 that had high experimentally measured cognate affinity (des1, des2, des3, des3.5, and wt2), all cognate and non-cognate colE/Im pairs were modeled in Rosetta by rigid-body docking and side-chain optimization as described above in the colE/Im interface design section.

### Interaction networks

In a selected set of designs, the steady-state fractional occupancy of each colE/Im interaction was calculated in ligand concentration that is equal to the experimentally determined cognate *K*_D_. For the Im interaction networks plotted in Fig. 8, the cognate was defined relative to the Im of each pair. The width of the line that represents each interaction is proportional to the fractional occupancy (values below 1% occupancy were neglected). The network specificity parameter *α* was calculated for each Im within the network as described above, given a colE concentration that is equal to the cognate interaction *K*_D_. Scripts for generating interaction networks between selected sets of designed pairs as well as all possible interaction networks are provided in Supplementary Data 2.

### Plasmids and bacterial strains

pET21d plasmid harboring colE^wt2^ gene followed by Im^wt2^ gene (separated by a 2-bp frameshift) with a C-terminal His_6_-tag^41^ was used as basis for cloning. The 59 cognate colE/Im designs were ordered from Gen9 Inc. (Cambridge, MA). The genes were ligated into a linearized pET21d plasmid using *Nco*I and *Xho*I restriction sites, transformed into T7 Express *lysY/I*^*q*^ *E.coli* cells (NEB), and five colonies of each design were sequenced. Designs with at least one colony that contained the designed colE/Im sequence were considered potentially active and forming the designed complex. Viability was also tested by transformation to the BL21 DE3 *E. coli* cells. In order to purify the designs, the endonuclease His127Ala inactivation mutation^48^ was introduced by QuickChange^75^. For Im expression, the Im gene with C-terminal His_6_-tag was cloned in the absence of the colE into pET21d plasmid.

### Protein expression and purification

BL21 (DE3) cultures were grown in Luria Broth (LB) medium at 37 ^°^C to OD_600_=0.6–0.8 and induced with 1 mM IPTG at 16 °C overnight. Cells were harvested and stored at −20 °C. Pellet was resuspended in 30 ml lysis buffer for 1 liter culture containing 50 mM Tris (pH 7.5), 50 mM NaCl, 10 mM imidazole and 1 mM MgCl_2_, sonicated and centrifuged as previously described^41^. The supernatant was loaded onto a column packed with 4 ml Ni-NTA beads for 1 liter culture, equilibrated with lysis buffer, washed with lysis buffer containing 20 mM imidazole, and eluted with lysis buffer containing 500 mM imidazole. For SPR, the colE was separated from the Im by dissociating the ColE from the His tagged Im with 6 M guanidine-HCl^23^ instead of lysis buffer, dialysis in water (×1000 v/v) followed by 50 mM phosphate buffer (pH 7.5). The colE was further purified on a cation-exchange column (SP HP; GE Healthcare) with a linear gradient to buffer containing 50 mM phosphate buffer (pH 7.5) and 1 M NaCl. The Im gene was expressed and purified on Ni-NTA as described above, followed by purification using gel filtration (HiLoad 16/600 Superdex 200 PG; GE Healthcare) equilibrated with 50 mM Tris (pH 7.5) and 150 mM NaCl. Before SPR, the colE and Im proteins were each dialyzed to buffer containing 50 mM MOPS (pH 7.5), 200 mM NaCl and 0.005% Tween-20. For crystallization, the colE/Im complex was co-expressed and purified as for Im alone, with the gel filtration buffer containing 50 mM Tris (pH 7.5) and 50 mM NaCl. The complex crystallized at a concentration above 70 mg/ml.

### Surface plasmon resonance

SPR experiments were performed using BIAcore T200 (GE Healthcare) at 25 ^°^C in buffer containing 50 mM MOPS (pH 7.5), 200 mM NaCl, and 0.005% Tween-20 (running buffer). ColE proteins were attached to CM5 chips (GE Healthcare) by amine coupling to a total of roughly 500 response units (RU). In the final stage of immobilization, the surfaces were blocked by 1 M ethanolamine (pH 8.0). Empty flow cells were used as concurrent negative controls. Im proteins were injected at 20 μl/min for 240–360 s association (depending on binding kinetics) followed by 720 s dissociation. A series of 8–12 Im concentrations was used, in most designs, using threefold dilutions starting from 18.9 μM, and in the remainder, twofold dilutions from starting concentrations that varied between 10 and 10,000 nM, depending on the design affinity. Regeneration was performed between cycles using 1–1.7 M guanidine hydrochloride. The data were analyzed using Biacore T200 evaluation software 3.0.

As a measure of confidence in the reported binding affinities, we chose 48 of the 81 pairs and repeated the SPR measurements exactly as explained above with freshly prepared reagents and SPR chips (repeat affinities are reported in parentheses in Supplementary Table 1). In all cases, the inferred dissociation constants exhibited less than tenfold differences between the repeats and mostly exhibited less than twofold differences (Supplementary Fig. 4). Furthermore, at the end of each series of cognate and non-cognate measurements for a given colE, we retested the designed-cognate Im protein at an intermediate concentration, and verified that the colE was still active and exhibited a similar binding response as at the start of the experiment.

Due to the vast heterogeneity in binding affinities and kinetics among designed pairs (at least six orders of magnitude), it was not possible to use a single fitting procedure to infer all of the dissociation constants. Specifically, all but two of the cognate dissociation constants and many of the high-affinity non-cognate ones were determined kinetically, by fitting the data to a single exponential or a two-state reaction model (Supplementary Fig. 3 and Supplementary Table 1). By contrast, for two cognate pairs (colE^des4^/Im^des4^ and colE^des8^/Im^des8^) and most of the non-cognate ones, kinetic models did not produce reliable fits for the data, and we therefore inferred the affinities using the steady-state analyte binding levels (*R*_eq_) at different concentrations (Fig. 3d and Supplementary Table 1). The *K*_D_ values for the repeat measurements were obtained using the same fitting procedure. For comparison, Fig. 3d and Supplementary Fig. 5 present all the cognate and non-cognate interactions using affinity fitting, including for the interactions that were determined kinetically.

### Structure determination and refinement

Crystals of colE^des3^/Im^des3^ and colE^des7^/Im^des7^ were obtained using the sitting-drop vapor-diffusion method with a Mosquito robot (TTP LabTech). Crystals of colE^des3^/Im^des3^ were grown from 25% PEG 200, 50 mM sodium phosphate dibasic/citric acid pH=4.2 and 100 mM NaCl. The crystals formed in the orthorhombic space group *C222*_*1*_, with two copies per asymmetric unit. A complete dataset to 2.25 Å resolution was collected at 100 K on a single crystal on in-house RIGAKU RU-H3R X-ray. The crystals of colE^des7^/Im^des7^ were grown from 12% PEG 1500 and 0.05M MMT buffer pH=8.0 (mixing DL-malic acid, MES and Tris base in the molar ratios 1:2:2—dl-malic acid). The crystals formed in the orthorhombic space group *P*2_1_2_1_2_1_, with one complex per asymmetric unit. A complete dataset to 1.56 Å resolution was collected at 100 K on a single crystal on in-house RIGAKU RU-H3R X-ray.

Diffraction images of the colE^des3^/Im^des3^ and colE^des7^/Im^des7^ crystals were indexed and integrated using the Mosflm program^76^, and the integrated reflections were scaled using the SCALA program^77^. Structure factor amplitudes were calculated using TRUNCATE^78^ from the CCP4 program suite. The colE^des3^/Im^des3^ and colE^des7^/Im^des7^ structures were solved by molecular replacement with the program PHASER^79^. The model used to solve colE^des3^/Im^des3^ and colE^des7^/Im^des7^ structures was colE^wt2^/Im^wt2^ complex (PDB code 3U43).

All steps of atomic refinement of both structures were carried out with the CCP4/REFMAC5 program^80^ and by Phenix refine^81^. The models were built into *2mF*_obs_ − *DF*_calc_, and *mF*_obs_ − *DF*_calc_ maps by using the COOT program^82^. Details of the refinement statistics of colE^des3^/Im^des3^ and colE^des7^/Im^des7^ structures are described in Supplementary Table 4.

### Code availability

Rosetta is available free of charge to all academic users (http://www.rosettacommons.org). Rosetta git version 627f7dd22223c3074594934b789abb4f4e2e3b10 was used for all design simulations. All Rosetta modeling and design was done using RosettaScripts^66^ that are available with their command lines and flag files in Supplementary Data 2.

## Electronic supplementary material

Supplementary Information

Description of Additional Supplementary Files

Supplementary Data 1

Supplementary Data 2

Reporting Summary

## Data Availability

The amino acid sequences and the computed Rosetta scores of the 59 designs that were tested experimentally and the wild type are available in Supplementary Data 1. The coordinates of the designs colE^des3^/Im^des3^ and colE^des7^/Im^des7^ are available from the RCSB Protein Data Bank with accession codes 6ERE and 6ER6, respectively. Plasmids encoding the 18 successful designs and designed pair 3.5 were deposited in the AddGene repository (https://www.addgene.org/Sarel_Fleishman/).
