# Supplementary material for: Ultrahigh specificity in a network of computationally designed protein-interaction pairs
Source: Nat Commun. 2018 Dec 11;9:5286. doi: 10.1038/s41467-018-07722-9 (PMC6290019; doi:10.1038/s41467-018-07722-9)
Supplement: Supplementary file 1 — Supplementary Information [file 41467_2018_7722_MOESM1_ESM.pdf]

Ultrahigh specificity in a network of computationally designed protein-interaction pairs

Netzer et al

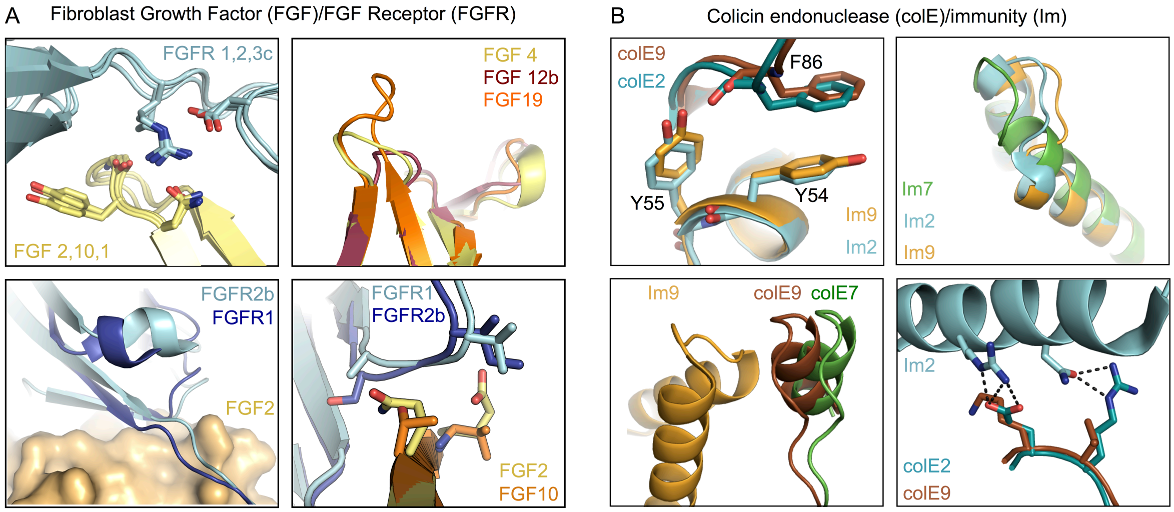

Supplementary Figure 1. The structural basis for affinity and specificity in natural protein-interaction networks. Upper left: conserved interaction hotspot contributes significantly to binding affinity. Upper right and lower panels: specificity determinants in interaction networks. Upper right: conformational changes and indels at interface loops. Lower left: rigid-body orientation changes between the binding partners in the different complexes. Lower right: Sequence changes at the interface of both binding partners. (A) Molecular representations were made based on PDB entries: 1CVS, 1NUN, 1RY7, 1IJT, 1Q1U, and 2P23. (B) PDB entries: 3U43, 1EMV, 7CEI, and 2WPT.

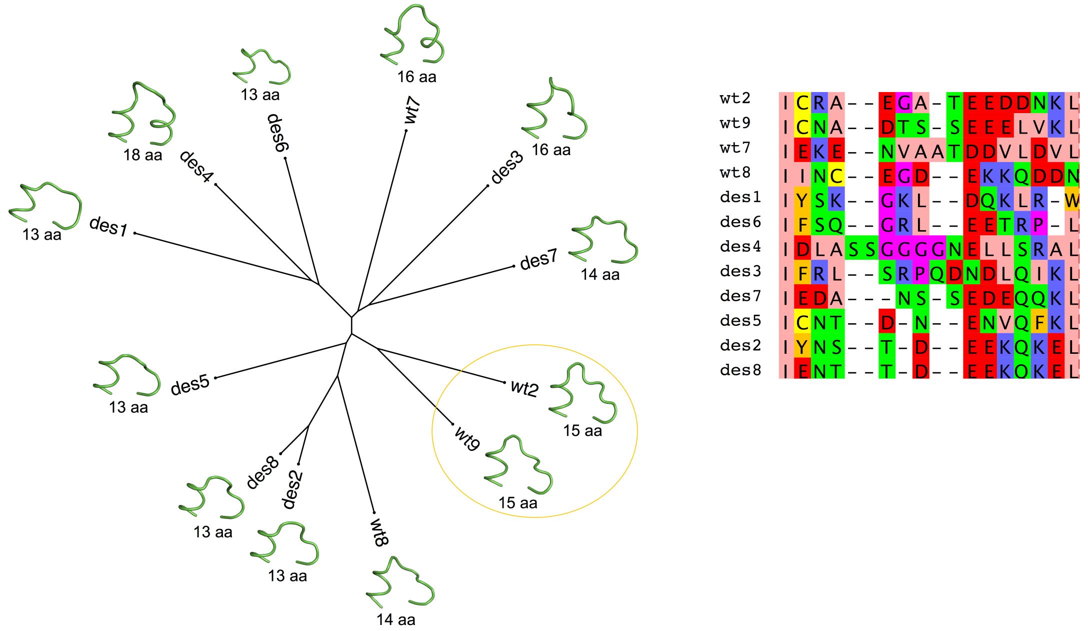

Supplementary Figure 2. Sequence and conformational diversity in designed loop I. Analysis of Im loop I sequences for the eight designs that were subjected to SPR indicates that most of the Im designs are more different in loop I than the natural colicin Im^{wt2} and Im^{wt9} (circled) are from one another. The designed sequences additionally contain many polar amino acids and indels**.** PDB entries for the wild type (wt) structures: 3U43, 1EMV, 7CEI, and 1GXH. Backbone conformations for the designed loop I's are extracted from the design models.

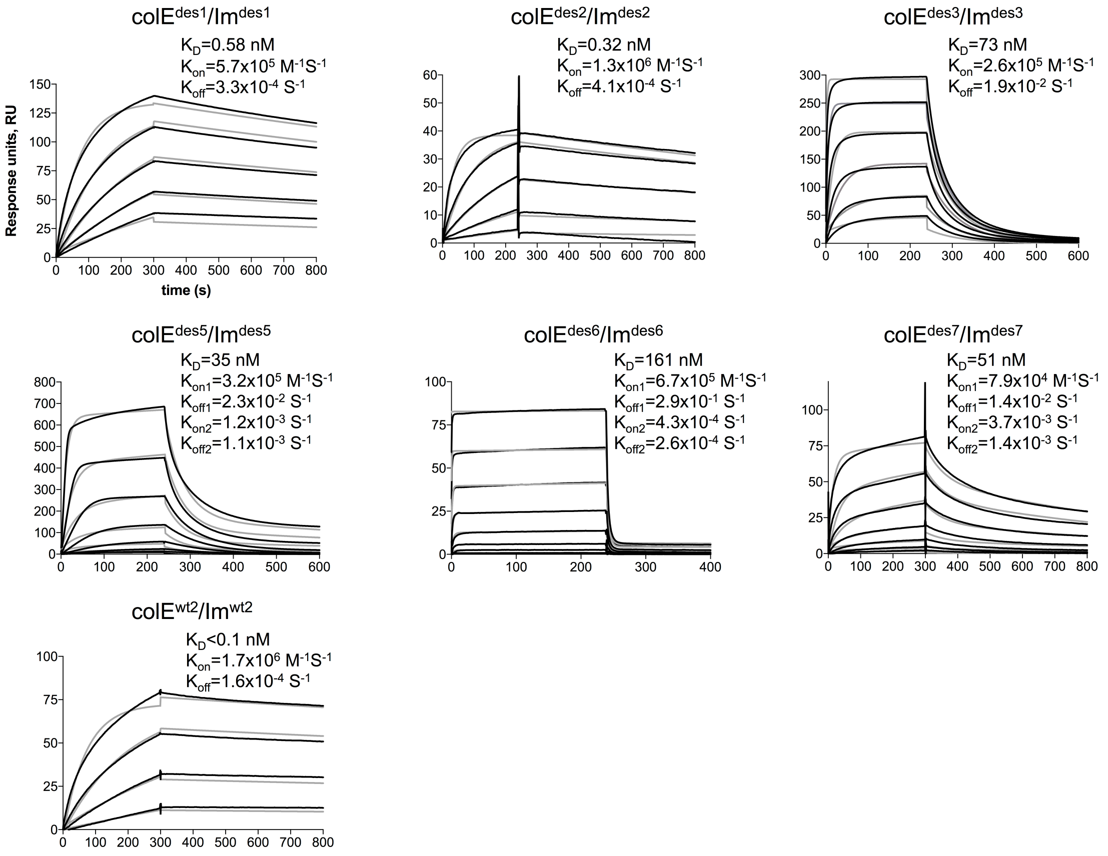

Supplementary Figure 3. SPR kinetic analysis of the binding of six cognate designed pairs and the wild type, related to Figure 3B. Data points shown in black and kinetic fits in gray. Ligand (Im) concentrations used for K_D analysis: des1: twofold dilutions from 25 nM; des2, des3, des5, des6, des7 and wt2: threefold dilutions from 25.9, 2,100, 233.3, 6,300, 700 and 8.6 nM, respectively. Interactions with single K_{on} and K_{off} values were fitted to single exponential and the others were fitted to two-state reaction.

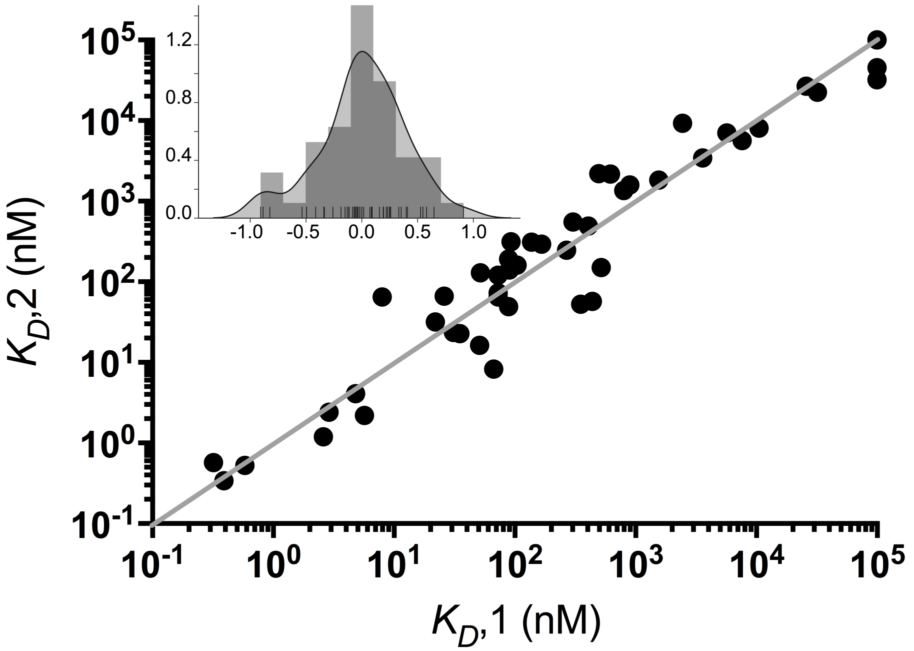

Supplementary Figure 4. Repeat SPR affinity measurements for 48 colE/Im interactions, related to Supplementary Table 1. The reported K_D values in Supplementary Table 1 (K_D,1) are plotted against their repeat measurements (K_D,2, values are provided in parentheses in Supplementary Table 1). y=x is plotted in grey. Inset: a histogram in log scale of the differences between the two measurements log($\frac{KD,2}{KD,1}$) shows that the majority of repeat measurements are within twofold.

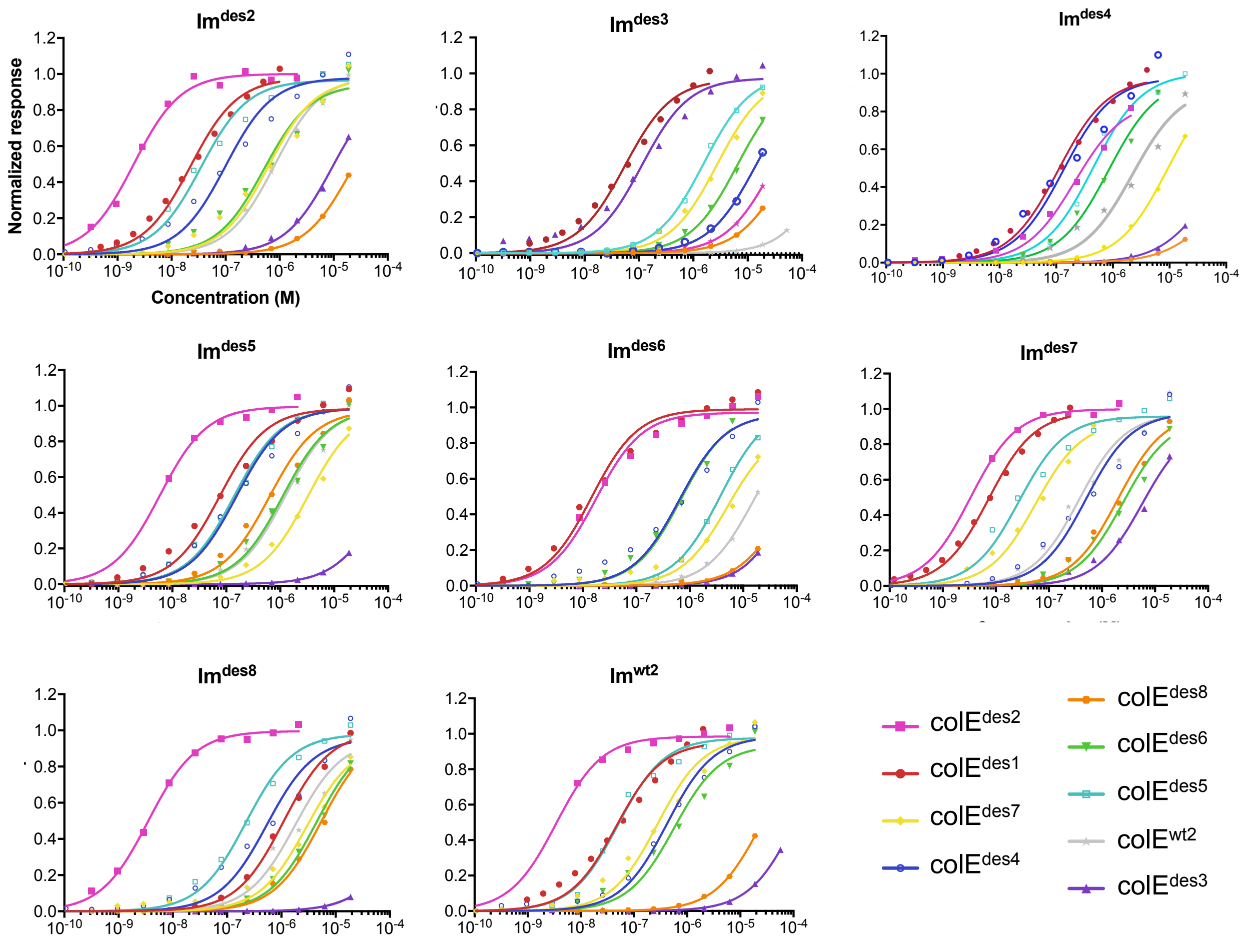

Supplementary Figure 5. Specificity of the designed Im proteins by SPR analysis to cognate and non-cognate colE proteins, related to figure 3D. K_D values in Supplementary Table 1. Binding of each Im to cognate and non-cognate colE proteins was measured using SPR at different Im concentrations and normalized to the maximal response at saturating concentrations (R_{max}) extracted by affinity fitting. For interactions that were determined kinetically, the affinity data were used to generate these plots, and in all cases yielded K_D within the same order of magnitude compared to the kinetically determined K_D.

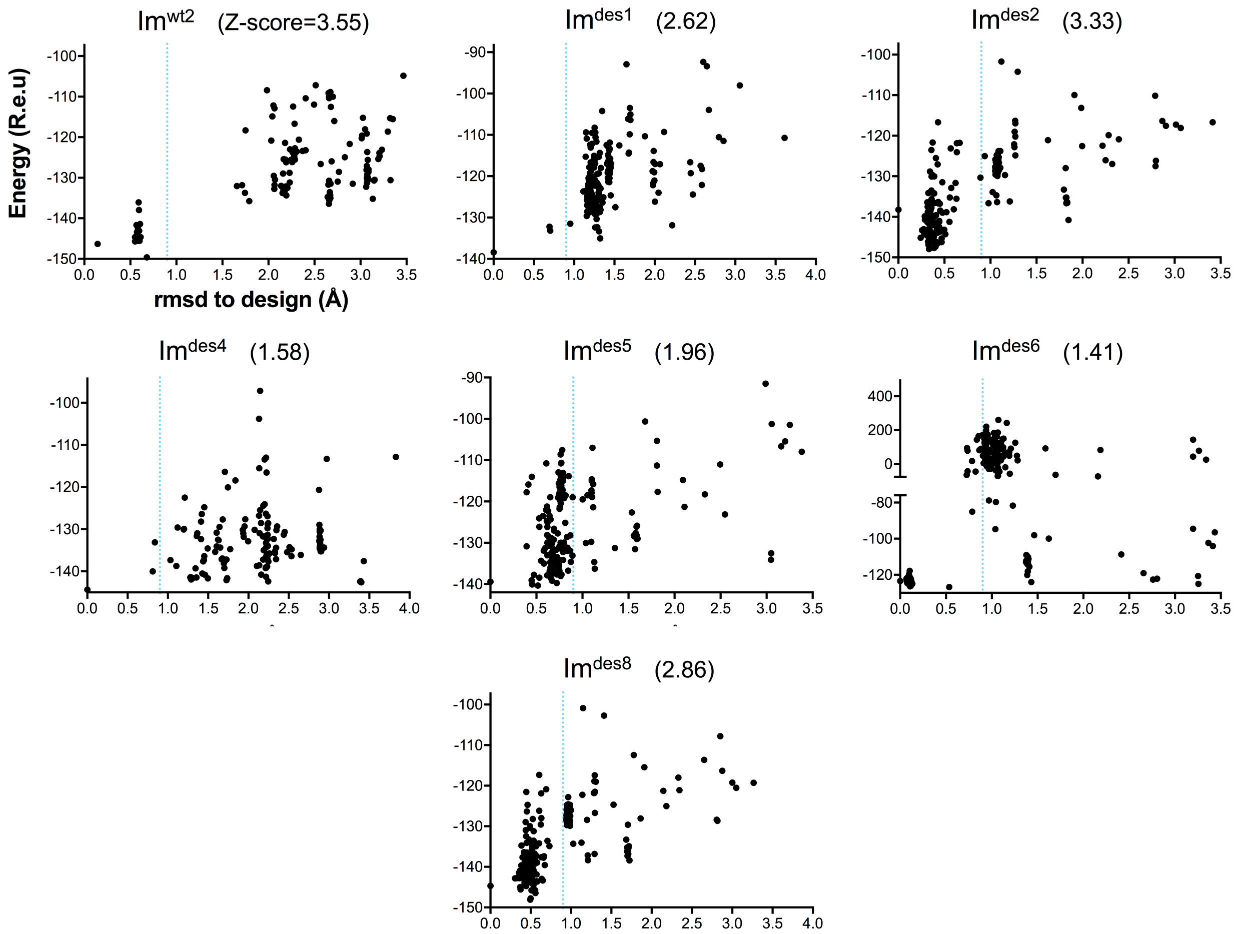

Supplementary Figure 6. Energy landscapes for loop I in the Im designs, related to Figure 6C. The sequence of each Im design was threaded on all Im backbone conformations of the same sequence length that were generated by the design algorithm (Fig. 1C). The energy of each model was calculated as well as its rmsd to the designed conformation to generate an energy landscape. Plots for Im^{des3} and Im^{des7} are in Fig 6C.

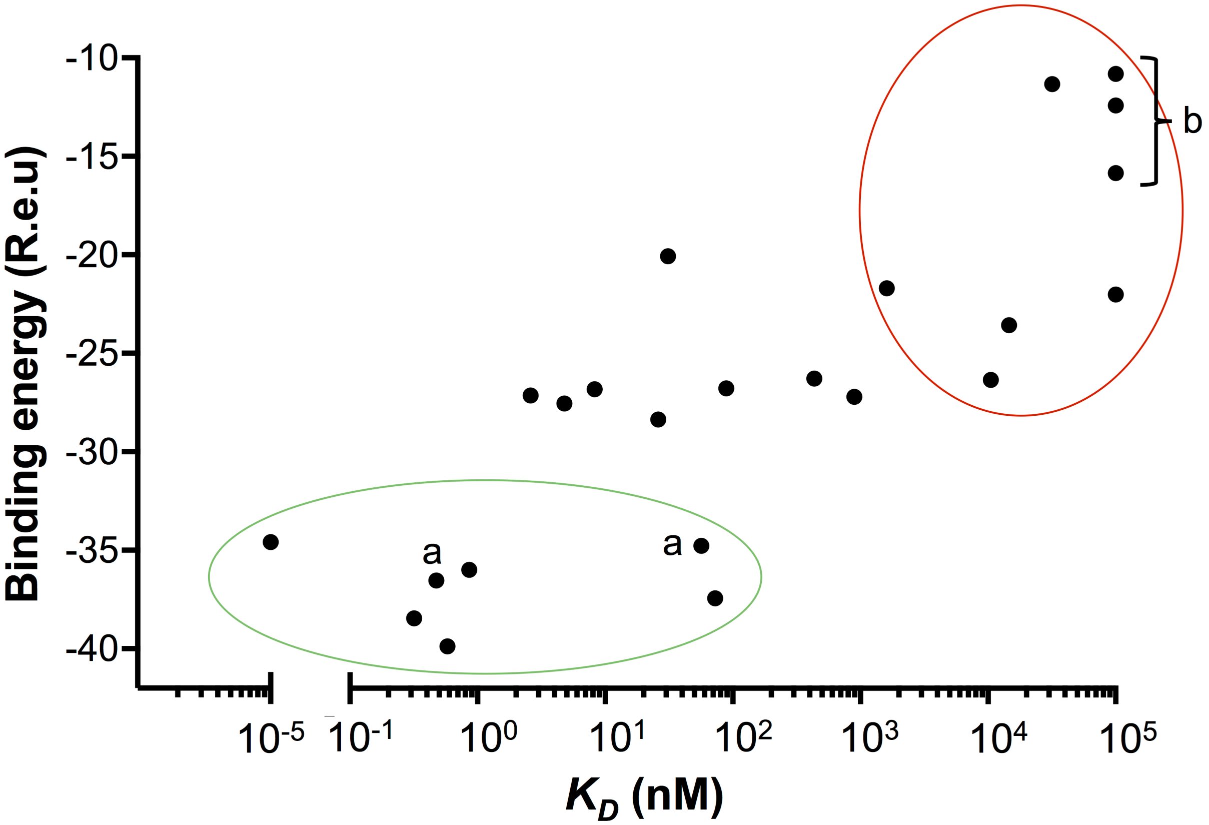

Supplementary Figure 7. Modeling discriminates cognate high-affinity colE/Im interactions from non-cognate low-affinity ones. Models of cognate and non-cognate colE/Im pairs with high predicted loop I preorganization and high experimentally measured cognate affinity (des1, des2, des3, des3.5, and wt2) were relaxed in Rosetta and their binding energies were plotted against experimentally determined K_Ds. Cognate pairs (green ellipse) have high computed and experimental affinity, including the related designs des3 and des3.5 cross-binding pairs (marked a). Non-cognate pairs, by contrast, which exhibited low affinity in experiments, were also predicted by Rosetta to bind weakly (red), particularly non-cognate pairs of $colE^{wt2}$ with Im^{des1}, Im^{des3}, and $Im^{des3.5}$ (marked b).

Supplementary Table 1: K_D values (nM) of cognate and non-cognate colE/Im interactions that were determined by SPR. Repeat measurements are noted in parentheses (see also Supplementary Fig. 4). For all interactions, an intermediate ligand concentration was repeated at the end of the experiment to verify the regeneration of the SPR chip.

$^{a}K_D$ values that were determined kinetically, fitting to single-exponential reactions

$^{b}K_D$ values that were determined kinetically, fitting to two-state reactions

All other values were determined using affinity fitting

^{c}Value taken from (Li et al. 2004).

N.B. No binding was detected in concentrations up to 6.3 μM

	Im^{des1}	Im^{des2}	Im^{des3}	$Im^{des3.5}$	Im^{des4}	Im^{des5}	Im^{des6}	Im^{des7}	Im^{des8}	Im^{wt2}
**$colE^{des1}$**	0.58^{a} (0.53)	4.8^{a} (4.1)	31^{a} (24)	-	138 (311)	22^{b} (32)	2.9^{a} (2.4)	0.39^{a} (0.34)	1,156	26^{b} (67)
**$colE^{des2}$**	89^{b} (49)	0.32^{a} (0.57)	31,900 (22,580)	-	268 (248)	8 (65)	67^{b} (8)	5.7^{b} (2.2)	1.8^{b}	2.6^{b} (1.2)
**$colE^{des3}$**	437^{a} (57)	10,530 (8,093)	73^{a} (73,65,121)	57^{a}	79,860	89,440	82,430	7,642 (5,663)	≥100,000 (32,000)	≥100,000 (45,680)
**$colE^{des3.5}$**	8.3^{a}	1609	0.48^{a}	0.86^{a}	-	-	-	949	4321	14,690
**$colE^{des4}$**	34	139	15,130	-	166 (294)	189	894	618 (2,180)	797 (1,360)	494 (2,192)
**$colE^{des5}$**	104^{b} (162)	89 (21)	1,557 (1,838)	-	405 (494)	35^{b} (23)	3,581 (3,435)	93 (313)	304 (552)	52 (130)
**$colE^{des6}$**	545^{b}	299^{b}	6936	-	962	1,265	161^{b}	3,426	4,848	148^{b}
**$colE^{des7}$**	2,189	90^{b} (141)	2,954	-	9,481	3,552	8,097	51^{b} (16)	4,121	351 (53)
**$colE^{des8}$**	7,684	24,540	57,090 (N.B.)	-	≥100,000 (N.B.)	812	73,790	2,293 (4,450)	5,696 (7,055)	25,860 (26,880)
**$colE^{wt2}$**	≥100,000 (≥100,000)	896 (1,586)	≥100,000 (≥100,000)	≥100,000	3,322	1,349	17,420	518 (151)	2,439 (9,284)	$<10^{-5\ c}$

Supplementary Table 2. The network specificity parameter α of each designed Im and colE protein to it cognate partner relative to all seven non-cognate designs. This value reflects the fraction of protein bound to its cognate partner relative to all non-cognate partners (Eq. 3). Since network specificity is concentration dependent, α was calculated for cognate and non-cognate at ligand concentrations of 1, 10, or 100 nM. Since cognate affinity varies substantially among the designs, we also calculated the normalized network specificity, in which the ligands are mixed at a concentration that is equal to the cognate interaction K_D (α_{KD}).

	des1	des2	des3	des4	des5	des6	des7	des8
Im α_{1nM}	11.75	3.71	0.42	0.40	0.17	0.023	0.022	0.00048
Im α_{10nM}	2.04	0.98	0.47	0.40	0.24	0.063	0.094	0.0019
Im α_{100nM}	0.46	0.42	0.65	0.37	0.39	0.24	0.23	0.012
Im α_{KD}	15.91	6.89	0.63	0.34	0.30	0.26	0.20	0.090
colE α_{1nM}	0.50	0.82	5.27	0.12	0.49	0.43	1.25	0.092
colE α_{10nM}	0.29	0.31	4.78	0.14	0.43	0.42	1.15	0.092
colE α_{100nM}	0.17	0.19	2.70	0.18	0.27	0.37	0.74	0.098
colE α_{KD}	0.53	1.38	3.06	0.19	0.35	0.34	0.88	0.19

Supplementary Table 3, related to Supplementary Figure 7. The experimental and computational binding energies of cognate and non-cognate colE/Im pairs that were predicted to have preorganized loop I (des1, des2, des3, des3.5, and wt2).
[a] The interaction K_D determined in SPR
[b] The computed binding affinity, in Rosetta energy units

Interaction pair	K_D (nM)[a]	Computed binding energy (R.e.u)[b]
$colE^{wt2}/Im^{wt2}$	0.00001	-34.571
$colE^{des2}/Im^{des2}$	0.32	-38.45
$colE^{des3.5}/Im^{des3}$	0.478	-36.514
$colE^{des1}/Im^{des1}$	0.58	-39.871
$colE^{des3.5}/Im^{des3.5}$	0.861	-35.973
$colE^{des2}/Im^{wt2}$	2.6	-27.129
$colE^{des1}/Im^{des2}$	4.8	-27.529
$colE^{des3.5}/Im^{des1}$	8.26	-26.804
$colE^{des1}/Im^{wt2}$	26	-28.343
$colE^{des1}/Im^{des3}$	31	-20.069
$colE^{des3}/Im^{des3.5}$	57	-34.755
$colE^{des3}/Im^{des3}$	73	-37.418
$colE^{des2}/Im^{des1}$	89	-26.771
$colE^{des3}/Im^{des1}$	437	-26.261

$colE^{wt2}/Im^{des2}$	896	-27.202
$colE^{des3.5}/Im^{des2}$	1,609	-21.678
$colE^{des3}/Im^{des2}$	10,530	-26.343
$colE^{des3.5}/Im^{wt2}$	14,690	-23.561
$colE^{des2}/Im^{des3}$	31,900	-11.331
$colE^{des3}/Im^{wt2}$	≥100,000	-22.013
$colE^{wt2}/Im^{des3}$	≥100,000	-10.794
$colE^{wt2}/Im^{des3.5}$	≥100,000	-12.401
$colE^{wt2}/Im^{des1}$	≥100,000	-15.835

Supplementary Table 4: Data collection and refinement statistics for colE^{des3}/Im^{des3} and colE^{des7}/Im^{des7}

	colE^{des7}/Im^{des7}	colE^{des3}/Im^{des3}
**Data Collection**		
PDB code	6ER6	6ERE
Space group	$P2_12_12_1$	$C222_1$
Cell dimensions:		
a,b,c (Å)	39.34, 56.30, 102.00	63.84, 113.37, 117.30
α,β,γ (˚)	90, 90, 90	90, 90, 90
No. of copies in a.u.	1	2
Resolution (Å)	50.00-1.56	55.63-2.25
Upper resolution shell (Å)	1.59-1.56	2.37-2.25
Unique reflections	32,776 (1,392)	20,578 (2,005)
Completeness (%)	99.2 (86.8)	98.8 (99.4)
Multiplicity	6.5 (6.0)	3.4 (3.3)
Average I/σ(I)	39.7 (4.9)	6.9 (2.4)
Rsym (I) (%)	5.1 (46.1)	13.3 (46.4)
**Refinement**		
Resolution range (Å)	25.5-1.56	40.76-2.25
No. of reflections (I/σ(I) > 0)	32,715	20,548
No. of reflections in test set	1,640	992
R-working (%) / R-free (%)	20.0 / 22.1	20.3/ 23.3
No. of protein atoms	1728	3422
No. of water molecules	238	41
Overall average B factor (Å^2)	36.21	31.01
Root mean square deviations:		
- bond length (Å)	0.009	0.015
- bond angle (°)	1.14	1.71
**Ramachandran Plot**		
Most favored (%)	98.6	97.8
Additionally allowed (%)	1.4	1.7
Disallowed (%)	1.1	0.0
