# Supplementary material for: Ultrahigh specificity in a network of computationally designed protein-interaction pairs
Source: Nat Commun. 2018 Dec 11;9:5286. doi: 10.1038/s41467-018-07722-9 (PMC6290019; doi:10.1038/s41467-018-07722-9)
Supplement: Supplementary file 2 — Description of Additional Supplementary Files [file 41467_2018_7722_MOESM2_ESM.pdf]

Description of Additional Supplementary Files

File Name: Supplementary Data 1
Description: Computational, experimental and sequence data on the colE/Im designs.

File Name: Supplementary Data 2
Description: Rosetta design protocols and scripts for generating interaction networks between selected sets of design pairs.
