# Supplementary material for: Ultrahigh specificity in a network of computationally designed protein-interaction pairs
Source: Nat Commun. 2018 Dec 11;9:5286. doi: 10.1038/s41467-018-07722-9 (PMC6290019; doi:10.1038/s41467-018-07722-9)
Supplement: Supplementary file 3 — Supplementary Data 1 [file 41467_2018_7722_MOESM3_ESM.docx]

**Supplemental Data 1. Computational, experimental and sequence data on the 59 colE/Im designs and colE^wt2^/Im^wt2^.**

^a^ Side-chains between (including) loop I stem, defined by alignment to Im^wt2^ Ile22 and Leu36 stem.

^b^ Packing quality at the E-Im core and interface, computed using RosettaHoles (Packstat) algorithm [(Sheffler and Baker, 2009)](https://paperpile.com/c/Bcxgs7/hQb1)

^c^ Solvent-Accessible Surface Area (SASA, Å^2^) buried upon complex formation

^d^ Interface shape complementarity (*Sc*) [(Lawrence and Colman, 1993)](https://paperpile.com/c/Bcxgs7/CuFA)

^e^ number of mutations on colE and Im combined relative to colE^wt2^/Im^wt2^

| **Design** | **Loop I length**^a^ | **Computational scores** | | | | **Viability in T7** | **AA sequence** | | **# mutations from wt2**^e^ |
| --- | --- | --- | --- | --- | --- | --- | --- | --- | --- |
|  |  | **Packstat**^b^ | ΔSASA^c^ | ***Sc***^d^ | **Binding energy** |  | **Im** | **colE** |  |
| wt2 | 15 | 0.72 | 1744 | 0.70 | -40.5 | + | MELKHSISDYTEAEFLEFVKKICRAEGATEEDDNKLVREFERLTEHPDGSDLIYYPRDDREDSPEGIVKEIKEWRAANGKSGFKQGLEHHHHHH | MESKRNKPGKATGKGKPVGDKWLDDAGKDSGAPIPDRIADKLRDKEFKNFDDFRKKFWEEVSKDPDLSKQFKGSNKTNIQKGKAPFARKKDQVGGRERFELHHDKPISQDGGVYDMNNIRVTTPKRHIDIHRGK | 0 |
| des1 | 13 | 0.68 | 1707 | 0.71 | -41.6 | + | MELKHSISDYTEAEFLEFVKKIYSKGKLDQKLRWVEEFVRLTEHPSGSDLIYYPRDDREDSPEGIVKEIKEWRAANGKSGFKQGLEHHHHHH | MESKRNKPGKATGKGKPVGDKWLDDAGKDSGAPIPDRIADKLRDKEFKNFDDFRKKFWEEVAKDPDLAKQFSRANQNEIKQGYAPFARQKDQVGGRRTYELHHDKPISQDGGVYDMNNIRVTTPKRHIDIHRGK | 30 |
| des2 | 13 | 0.69 | 1803 | 0.69 | -40.4 | + | MELKHSISDYTEAEFLEFVKKIYNSTDEEKQKELVTEFERLTEHPSGSDLIYYPRDDREDSPEGIVKEIKEWRAANGKSGFKQGLEHHHHHH | MESKRNKPGKATGKGKPVGDKWLDDAGKDSGAPIPDRIADKLRDKEFKNFDDFRKKFWKEVAKDPDLAKQFKRANRRNIKQGRAPFAPKKDQVGGRRTFELHHDKPISQDGGVYDMNNIRVTTPKRHIDIHRGK | 26 |
| des3 | 16 | 0.66 | 1615 | 0.72 | -39.0 | + | MELKHSISDYTEAEFLEFVKDIFRLSRPQDNDLQIKLVLEFKRLTEHPDGSDLIYYPRSDREDSPEGIVKEIKEWRAANGKSGFKQGLEHHHHHH | MESKRNKPGKATGKGKPVGDKWLDDAGKDSGAPIPDRIADKLRDKEFKNFDDFRKKFWKEVAKDPDLAKQFSKANQRNIKDGNAPFARESDQVGGRTTYELHHDKPISQDGGVYDMNNIRVTTPKRHIDIHRGK | 31 |
| des4 | 18 | 0.73 | 1871 | 0.72 | -40.0 | + | MELKHSISDYTEAEFLEFVKKIDLASSGGGGNELLSRALVEEFERLTEHPDGSDLIYYPRDDREDSPEGIVKEIKEWRAANGKSGFKQGLEHHHHHH | MESKRNKPGKATGKGKPVGDKWLDDAGKDSGAPIPDRIADKLRDKEFKNFDDFRKKFWEEVSKDPDLSKQFKRANKDSISKGQAPFARKKDQVGGRKTFELHHDKPISQDGGVYDMNNIRVTTPKRHIDIHRGK | 22 |
| des5 | 13 | 0.65 | 1605 | 0.71 | -35.7 | + | MELKHSISDYTEAEFLEFVKKICNTDNENVQFKLVTEFERLTEHPDGSDLIYYPRDDREDSPEGIVKEIKEWRAANGKSGFKQGLEHHHHHH | MESKRNKPGKATGKGKPVGDKWLDDAGKDSGAPIPDRIADKLRDKEFKNFDDFKKKFWKEVAKDPDLSKQFKGSNQKNIKNGQAPFARQKDQVGGRRRFELHHDKPISQDGGVYDMNNIRVTTPKRHIDIHRGK | 21 |
| des6 | 13 | 0.70 | 1585 | 0.73 | -36.6 | + | MELKHSISDYTEAEFLEFVKKIFSQGRLEETRPLVEEFERLTEHPDGSDLVYYPRDDREDSPEGIVKEIKEWRAANGKSGFKQGLEHHHHHH | MESKRNKPGKATGKGKPVGDRWLDDAGKDSGAPIPDRIADKLRDKEFKNFDDFRKKFWEEVSKDPDLAKQFNRANREHIKQGQAPFAPKKNQVGGRQTFELHHDKPISQDGGVYDMNNIRVTTPKRHIDIHRGK | 27 |
| des7 | 14 | 0.69 | 1750 | 0.69 | -45.7 | + | MELKHSISDYTEAEFLEFVKKIEDANSSEDEQQKLVEEFIRLTEHPSGSDLIYYPRDDREDSPEGIVKEIKEWRAANGKSGFKQGLEHHHHHH | MESKRNKPGKATGKGKPVGDKWLDDAGKDSGAPIPDRIADKLRDKEFKNFDDFRKKFWEEVSKDPDLAKQFKRSNRKRIQQGYAPFAPQKDQVGGRTTFELHHDKPISQDGGVYDMNNIRVTTPKRHIDIHRGK | 24 |
| des8 | 13 | 0.72 | 1655 | 0.71 | -42.6 | + | MELKHSISDYTEAEFLEFVKKIENTTDEEKQKELVEEFERLTEHPSGSDLVYYPRDDREDSPEGIVKEIKEWRAANGKSGFKQGLEHHHHHH | MESKRNKPGKATGKGKPVGDKWLDDAGKDSGAPIPDRIADKLRDKEFKNFDDFRKKFWEEVSKDPDLAKQFNRQNKKNIKQGQAPFAPQKDQVGGRKTFELHHDKPISQDGGVYDMNNIRVTTPKRHIDIHRGK | 25 |
| des9 | 13 | 0.65 | 1627 | 0.70 | -44.7 | + | MELKHSISDYTEAEFLEFVKKIDELTHEEHQAQLVDEFERLTEHPDGSDLVYYPRDDREDSPEGIVKEIKEWRAANGKSGFKQGLEHHHHHH | MESKRNKPGKATGKGKPVGDKWLDDAGKDSGAPIPDRIADKLRDKEFKNFDDFRKKFWEEVSKDPDLSKQFKRSNQENIKQGQAPFARQKDQVGGRRVFELHHDKPISQDGGVYDMNNIRVTTPKRHIDIHRGK | 21 |
| des10 | 13 | 0.71 | 1678 | 0.68 | -43.8 | + | MELKHSISDYTEAEFLEFVKKIIATNDETQQRALVDEFERLTEHPDGSDLIYYPRDDREDSPEGIVKEIKEWRAANGKSGFKQGLEHHHHHH | MESKRNKPGKATGKGKPVGDKWLDDAGKDSGAPIPDRIADKLRDKEFKNFDDFRKKFWEEVSKDPDLSKQFKGANQDSIKQGQAPFARSKDQVGGRRTFELHHDKPISQDGGVYDMNNIRVTTPKRHIDIHRGK | 23 |
| des11 | 17 | 0.69 | 1708 | 0.71 | -37.7 | + | MELKHSISDYTEAEFLEFVKKICNLSAGNASVDEMEKAVDEFERLTEHPSGSDLIYYPRDDREDSPEGIVKEIKEWRAANGKSGFKQGLEHHHHHH | MESKRNKPGKATGKGKPVGDKWLDDAGKDSGAPIPDRIADKLRDKEFKNFDDFKKKFWEEVSKDPDLSKQFKGSNQESIKKGLAPFARQNDQVGGRRVFELHHDKPISQDGGVYDMNNIRVTTPKRHIDIHRGK | 24 |
| des12 | 17 | 0.72 | 1634 | 0.74 | -39.7 | + | MELKHSISDYTEAEFLEFVKEICQKNSAGELDESLFKLVTEFERLTEHPDGSDLIYYPRDDREDSPEGIVKEIKEWRAANGKSGFKQGLEHHHHHH | MESKRNKPGKATGKGKPVGDKWLDDAGKDSGAPIPDRIADKLRDKEFKNFDDFKKKFWKEVSKDPDLSKQFKGSNRENIKQGIAPFARQKDQVGGRRVFELHHDKPISQDGGVYDMNNIRVTTPKRHIDIHRGK | 22 |
| des13 | 13 | 0.67 | 1775 | 0.71 | -42.5 | + | MELKHSISDYTEAEFLEFVKKIMASNDETQQRKLVTEFERLTEHPDGSDLIYYPRDDREDSPEGIVKEIKEWRAANGKSGFKQGLEHHHHHH | MESKRNKPGKATGKGKPVGDKWLDDAGKDSGAPIPDRIADKLRDKEFKNFDDFRKKFWEEVSKDPDLSKQFKRSNQNNIKQGIAPFARSKDQVGGRRTFELHHDKPISQDGGVYDMNNIRVTTPKRHIDIHRGK | 21 |
| des14 | 15 | 0.69 | 1706 | 0.70 | -39.1 | + | MELKHSISDYTEAEFLEFVKKICEGHFEAGSDGPKVVQEFERLTEHPDGSDLIYYPRDDREDSPEGIVKEIKEWRAANGKSGFKQGLEHHHHHH | MESKRNKPGKATGKGKPVGDKWLDDAGKDSGAPIPDRIADKLRDKEFKNFDDFKRKFWEEVSKDPDLSKQFKGSNQNRIKQGQAPFARKKDQVGGRKTFELHHDKPISQDGGVYDMNNIRVTTPKRHIDIHRGK | 22 |
| des15 | 13 | 0.68 | 1709 | 0.63 | -40.8 | + | MELKHSISDYTEAEFLEFVKEILEIQDEELQKIQVEEFERLTEHPDGSDLIYYPRDDRDDSPEGIVKEIKEWRAANGKSGFKQGLEHHHHHH | MESKRNKPGKATGKGKPVGDKWLDDAGKDSGAPIPDRIADKLRDKEFKNFDDFRKKFWEEVSKDPDLSKQFKGANQRNIKQGQAPFAPKKDQVGGRKTFELHHDKPISQDGGVYDMNNIRVTTPKRHIDIHRGK | 23 |
| des16 | 16 | 0.69 | 1632 | 0.57 | -33.3 | + | MELKHSISDYTEAEFLEFVKKSCEAELEGREEVAKRLVEEFERLTEHPDGSDLIYYPRDDREDSPEGIVKEIKEWRAANGKSGFKQGLEHHHHHH | MESKRNKPGKATGKGKPVGDKWLDDAGKDSGAPIPDRIADKLRDKEFKNFDDFKKKFWKEVSKDPDLSKQFKGSNKLSISQGQAPFARQKDQVGGRRRFELHHDKPISQDGGVYDMNNIRVTTPKRHIDIHRGK | 20 |
| des17 | 12 | 0.71 | 1707 | 0.67 | -45.2 | + | MELKHSISDYTEAEFLEFVKKICKADEEQARQLVEEFIRLTEHPSGSDLVYYPRDDREDSPEGIVKEIKEWRAANGKSGFKQGLEHHHHHH | MESKRNKPGKATGKGKPVGDKWLDDAGKDSGAPIPDRIADKLRDKEFKNFDDFRKKFWEEVSKDPDLAKQFKRSNRDRIKQGQAPFAPQKDQVGGRKTFELHHDKPISQDGGVYDMNNIRVTTPKRHIDIHRGK | 25 |
| des18 | 10 | 0.70 | 1585 | 0.73 | -36.6 | + | MELKHSISDYTEAEFLEFVKKIVEGLLGQPSVEEFIRLTEHPSGSDLIYYPRDDREDSPEGIVKEIKEWRAANGKSGFKQGLEHHHHHH | MESKRNKPGKATGKGKPVGDRWLDDAGKDSGAPIPDRIADKLRDKEFKNFDDFRKKFWEEVSKDPDLAKQFKRANRNRIKQGEAPFAPQKDQVGGRKTFELHHDKPISQDGGVYDMNNIRVTTPKRHIDIHRGK | 29 |
| des19 | 16 | 0.70 | 1611 | 0.71 | -37.8 | - | MELKHSISDYTEAEFLEFVKKIWHMRGANFLDKQIQLVLEFERLTEHPDGSDLIYYPRSDREDSPEGIVKEIKEWRAANGKSGFKQGLEHHHHHH | MESKRNKPGKATGKGKPVGDKWLDDAGKDSGAPIPDRIADKLRDKEFKNFDDFRKKFWEEVAKDPDLAKQFNPQNQDNIKNGYAPFARQSDQVGGRTTFELHHDKPISQDGGVYDMNNIRVTTPKRHIDIHRGK | 27 |
| des20 | 13 | 0.73 | 1592 | 0.69 | -42.0 | - | MELKHSISDYTEAEFLEFVKKIEQSNSEEKQRILVEEFERLTEHPDGSDLIYYPRDDREDSPEGIVKEIKEWRAANGKSGFKQGLEHHHHHH | MESKRNKPGKATGKGKPVGDKWLDDAGKDSGAPIPDRIADKLRDKEFKNFDDFRKKFWEEVSKDPDLSKQFKRANKASIKQGQAPFARSKDQVGGRRTFELHHDKPISQDGGVYDMNNIRVTTPKRHIDIHRGK | 29 |
| des21 | 12 | 0.71 | 1710 | 0.69 | -46.5 | - | MELKHSISDYTEAEFLEFVKKIQHSPERTQEAMVTEFERLTEHPDGSDLIYYPRDDREDSPEGIVKEIKEWRAANGKSGFKQGLEHHHHHH | MESKRNKPGKATGKGKPVGDKWLDDAGKDSGAPIPDRIADKLRDKEFKNFDDFRKKFWEEVSKDPDLSKQFNRSNKENIKRGWAPFARQKDQVGGRRVFELHHDKPISQDGGVYDMNNIRVTTPKRHIDIHRGK | 22 |
| des22 | 16 | 0.69 | 1722 | 0.72 | -41.7 | - | MELKHSISDYTEAEFLEFVKKIDRAVSNGNEDLARKLVDEFERLTEHPDGSDLVYYPRDDREDSPEGIVKEIKEWRAANGKSGFKQGLEHHHHHH | MESKRNKPGKATGKGKPVGDKWLDDAGKDSGAPIPDRIADKLRDKEFKNFDDFREKFWKEVSKDPDLSKQFKGSNRKSIKQGRAPFARRKDQVGGRRTFELHHDKPISQDGGVYDMNNIRVTTPKRHIDIHRGK | 22 |
| des23 | 14 | 0.68 | 1630 | 0.65 | -36.9 | - | MELKHSISDYTEAEFLEFVKKIIKLNAQGVYTPVNVTEFERLTEHPDGSDLIYYPRDDREDSPEGIVKEIKEWRAANGKSGFKQGLEHHHHHH | MESKRNKPGKATGKGKPVGDKWLDDAGKDSGAPIPDRIADKLRDKEFKNFDDFRKKFWKEVSKDPDLSKQFKGANKESIKKGQAPFARKNDQVGGRRVFELHHDKPISQDGGVYDMNNIRVTTPKRHIDIHRGK | 24 |
| des24 | 13 | 0.71 | 1654 | 0.66 | -37.7 | - | MELKHSISDYTEAEFLEFVKKILKVPIEDQNKKLVEEFERLTEHPDGSDLIYYPRDDREDSPEGIVKEIKEWRAANGKSGFKQGLEHHHHHH | MESKRNKPGKATGKGKPVGDKWLDDAGKDSGAPIPDRIADKLRDKEFKNFDDFRKKFWEEVSKDPDLSKQFKGSNKLRIKQGQAPFAPKKDQVGGRKVFELHHDKPISQDGGVYDMNNIRVTTPKRHIDIHRGK | 20 |
| des25 | 17 | 0.70 | 1679 | 0.71 | -37.6 | - | MELKHSISDYTEAEFLEFVKEIIQQLRSGTTSTDLQPEVTEFERLTEHPSGSDLIYYPRDDREDSPEGIVKEIKEWRAANGKSGFKQGLEHHHHHH | MESKRNKPGKATGKGKPVGDKWLDDAGKDSGAPIPDRIADKLRDKEFKNFDDFKKKFWKEVAKDPDLSKQFKGSNQDSIKQGLSPFAPQKDQVGGRRVFELHHDKPISQDGGVYDMNNIRVTTPKRHIDIHRGK | 30 |
| des26 | 16 | 0.72 | 1700 | 0.72 | -43.0 | - | MELKHSISDYTEAEFLEFVKKIIEGSGQNSEEEAIKLVEEFIRLTEHPDGSDLIYYPRDDREDSPEGVVKEIKEWRAANGKSGFKQGLEHHHHHH | MESKRNKPGKATGKGKPVGDKWLDDAGKDSGAPIPDRIADKLRDKEFKNFDDFRKKFWKEVAKDPDLSKQFKGANKESIRQGIAPFARENDQVGGRRVFELHHDKPISQDGGVYDMNNIRVTTPKRHIDIHRGK | 25 |
| des27 | 13 | 0.69 | 1751 | 0.62 | -38.2 | - | MELKHSISDYTEAEFLEFVKKIIATEDETQRQALVEEFERLTEHPDGSDLIYYPRDDREDSPEGIVKEIKEWRAANGKSGFKQGLEHHHHHH | MESKRNKPGKATGKGKPVGDKWLDDAGKDSGAPIPDRIADKLRDKEFKNFDDFRKKFWEEVSKDPDLSKQFKRANQDSIKKGQAPFARSKDTVGGRRTFELHHDKPISQDGGVYDMNNIRVTTPKRHIDIHRGK | 23 |
| des28 | 14 | 0.70 | 1847 | 0.74 | -38.7 | - | MELKHSISDYTEAEFLEFVKKICQGNREGQPLFQAVTEFERLTEHPDGSDLIYYPRDDREDSPEGIVKEIKEWRAANGKSGFKQGLEHHHHHH | MESKRNKPGKATGKGKPVGDKWLDDAGKDSGAPIPDRIADKLRDKEFKNFDDFRKKFWKEVAKDPDLSKQFKRSNQESIKKGLAPYARRKDQVGGRRIFELHHDKPISQDGGVYDMNNIRVTTPKRHIDIHRGK | 25 |
| des29 | 20 | 0.66 | 1678 | 0.72 | -40.0 | - | MELKHSISDYTEAEFLEFVKKICSINAQGSSEQQEQKLPDMVSEFERLTEHPSGSDLIYYPRDDREDSPEGIVKEIKEWRAANGKSGFKQGLEHHHHHH | MESKRNKPGKATGKGKPVGDKWLDDAGKDSGAPIPDRIADKLRDKEFKNFDDFRKKFWEEVSKDPDLSKQFKGANQASIKQGYSPFAPQKDQVGGRRTFELHHDKPISQDGGVYDMNNIRVTTPKRHIDIHRGK | 27 |
| des30 | 18 | 0.67 | 1805 | 0.69 | -41.4 | - | MELKHSISDYTEAEFLEFVKKILDQNALNPTAISLNIKLVTEFERLTEHPDGSDLIYYPRDDREDSPEGIVKEIKEWRAANGKSGFKQGLEHHHHHH | MESKRNKPGKATGKGKPVGDKWLDDAGKDSGAPIPDRIADKLRDKEFKNFDDFRKKFWEEVAKDPDLSKQFKGSNRLSIKSGQAPFARQKDQVGGRRVFELHHDKPISQDGGVYDMNNIRVTTPKRHIDIHRGK | 23 |
| des31 | 20 | 0.71 | 1946 | 0.61 | -42.1 | - | MELKHSISDYTEAEFLEFVKKIQTKNRLSHLPPDYEGEQKLVEEFERLTEHPDGSDLIYYPREDREDSPEGIVKEIKEWRAANGKSGFKQGLEHHHHHH | MESKRNKPGKATGKGKPVGDKWLDDAGKDSGAPIPDRIADKLRDKEFKNFDDFRKKFWEEVSKDPDLSKQFQGSNQTRISKGQAPFARTKDTVGGRKTFELHHDKPISQDGGVYDMNNIRVTTPKRHIDIHRGK | 27 |
| des32 | 17 | 0.70 | 1856 | 0.67 | -42.9 | - | MELKHSISDYTEAEFLEFVKKICDHHGDAQLEALNQKEVEEFERLTEHPDGSDLIYYPRDDREDSPEGIVKEIKEWRAANGKSGFKQGLEHHHHHH | MESKRNKPGKATGKGKPVGDKWLDDAGKDSGAPIPDRIADKLRDKEFKNFDDFRKKFWEEVAKDPDLSKQFKGANQRSIKQGQSPFARKKDQVGGRRTFELHHDKPISQDGGVYDMNNIRVTTPKRHIDIHRGK | 25 |
| des33 | 15 | 0.70 | 1919 | 0.74 | -43.1 | - | MELKHSISDYTEAEFLEFVKKITNFNSNTEVLSEELVTEFERLTEHPDGSDLIYYPREDRDDSPEGIVKEIKEWRAANGKSGFKQGLEHHHHHH | MESKRNKPGKATGKGKPVGDKWLDDAGKDSGAPIPDRIADKLRDKEFKNFDDFRKKFWEEVSKDPDLAKQFKGGNQRRIKQGQAPFARKNDQVGGRRRFELHHDKPISQDGGVYDMNNIRVTTPKRHIDIHRGK | 24 |
| des34 | 19 | 0.71 | 1642 | 0.69 | -36.8 | - | MELKHSISDYTEAEFLEFVKEITKYVEGKTSMSDETIDKLVEEFIRLTEHPSGSDLIYYPRDDREDSPEGIVKEIKEWRAANGKSGFKQGLEHHHHHH | MESKRNKPGKATGKGKPVGDKWLDDAGKDSGAPIPDRIADKLRDKEFKNFDDFRKKFWEEVAKDPDLAKQFSPNNRKNIKQGYAPFAPQKDQVGGRTRFELHHDKPISQDGGVYDMNNIRVTTPKRHIDIHRGK | 29 |
| des35 | 13 | 0.72 | 1676 | 0.70 | -34.9 | - | MELKHSISDYTEAEFLEFVKKICNSTDEEEARKLVEEFERLTEHPDGSDLVYYPRDDREGSPEGIVKEIKEWRAANGKSGFKQGLEHHHHHH | MESKRNKPGKATGKGKPVGDKWLDDAGKDSGAPIPDRIADKLRDKEFKNFDDFRKKFWEEVSKDPDLAKQFKPGNRDRIKQGQAPFARKNDQVGGRKTFELHHDKPISQDGGVYDMNNIRVTTPKRHIDIHRGK | 24 |
| des36 | 12 | 0.72 | 1781 | 0.67 | -40.9 | - | MELKHSISDYTEAEFLEFVKKILNLPAEDNRSLVREFIRLTEHPSGSDLIYYPRDDREDSPEGIVKEIKEWRAANGKSGFKQGLEHHHHHH | MESKRNKPGKATGKGKPVGDRWLDDAGKDSGAPIPDRIADKLRDKEFKNFDDFRKKFWEEVSKDPDLAKQFKGSNRKRIQQGKAPFAPQKDQVGGRETFELHHDKPISQDGGVYDMNNIRVTTPKRHIDIHRGK | 22 |
| des37 | 13 | 0.71 | 1661 | 0.76 | -40.8 | - | MELKHSISDYTEAEFLEFVKKILTLPTGKEQFDLVEEFIRLTEHPSGSDLIYYPRDDRDDSPEGIVKEIKEWRAANGKSGFKQGLEHHHHHH | MESKRNKPGKATGKGKPVGDRWLDDAGKDSGAPIPDRIADKLRDKEFKNFDDFRKKFWEEVSKDPDLAKQFKRSNRDRIKQGQAPFAPQKDQVGGRKVFELHHDKPISQDGGVYDMNNIRVTTPKRHIDIHRGK | 28 |
| des38 | 13 | 0.71 | 1692 | 0.69 | -42.1 | - | MELKHSISDYTEAEFLEFVKEIEKSTDADEQEKLVLEFERLTEHPSGSDLIYYPRDDREDSPEGIVKEIKEWRAANGKSGFKQGLEHHHHHH | MESKRNKPGKATGKGKPVGDKWLDDAGKDSGAPIPDRIADKLRDKEFKNFDDFRKKFWKEVSKDPDLSKQFNRNNQRNIKRGKAPFARKSDQVGGRTRFELHHDKPISQDGGVYDMNNIRVTTPKRHIDIHRGK | 23 |
| des39 | 16 | 0.66 | 1757 | 0.62 | -34.0 | - | MELKHSISDYTEAEFLEFVKKILKASRNGDEDTARKLVDEFKRLTEHPDGSDLIYYPRSDREDSPEGIVKEIKEWRAANGKSGFKQGLEHHHHHH | MESKRNKPGKATGKGKPVGDKWLDDAGKDSGAPIPDRIADKLRDKEFKNFDDFRRKFWKEVAKDPDLAKQFNEQNQGNIKKGNAPFARESDQVGGRRSFELHHDKPISQDGGVYDMNNIRVTTPKRHIDIHRGK | 28 |
| des40 | 12 | 0.74 | 1740 | 0.70 | -44.5 | - | MELKHSISDYTEAEFLEFVKKIEEAPGEDAQKMVEEFKRLTEHPDGSDLIYYPRSDREDSPEGIVKEIKEWRAANGKSGFKQGLEHHHHHH | MESKRNKPGKATGKGKPVGDKWLDDAGKDSGAPIPDRIADKLRDKEFKNFDDFRKKFWEEVAKDPDLAKQFNSQNQKRIKQGYAPFARESDQVGGRRRFELHHDKPISQDGGVYDMNNIRVTTPKRHIDIHRGK | 26 |
| des41 | 16 | 0.72 | 1758 | 0.73 | -45.7 | - | MELKHSISDYTEAEFLEFVKDIWSNQEELSEDQQRKIVTEFERLTEHPSGSDLIYYPRSDREDSPEGIVKEIKEWRAANGKSGFKQGLEHHHHHH | MESKRNKPGKATGKGKPVGDKWLDDAGKDSGAPIPDRIADKLRDKEFKNFDDFRKKFWKEVAKDPDLAKQFNPQNQKNIKAGYAPFARQSDQVGGRRTFELHHDKPISQDGGVYDMNNIRVTTPKRHIDIHRGK | 31 |
| des42 | 18 | 0.68 | 1904 | 0.75 | -45.3 | - | MELKHSISDYTEAEFLEFVKEIERLQDDESIPEETLRKLVEEFERLTEHPDGSDLIYYPRSDREDSPEGIVKEIKEWRAANGKSGFKQGLEHHHHHH | MESKRNKPGKATGKGKPVGDKWLDDAGKDSGAPIPDRIADKLRDKEFKNFDHFRKAFWKEVAKDPDLAKQFNPQNQKNIKAGLAPFARQSDQVGGRKTFELHHDKPISQDGGVYDMNNIRVTTPKRHIDIHRGK | 31 |
| des43 | 20 | 0.69 | 1779 | 0.68 | -37.8 | - | MELKHSISDYTEAEFLEFVKKIVAILEYGSSEQSDKYIPKLLDEFERLTEHPDGSDLIFYPRDDREDSPEGIVKEIKEWRAANGKSGFKQGLEHHHHHH | MESKRNKPGKATGKGKPVGDKWLDDAGKDSGAPIPDRIADKLRDKEFKNFDAFREAFWREVAKDPDLAKQFKRANQGNIKNGNAPFARESDQVGGRRRFELHHDKPISQDGGVYDMNNIRVTTPKRHIDIHRGK | 33 |
| des44 | 16 | 0.73 | 1719 | 0.72 | -39.5 | - | MELKHSISDYTEAEFLEFVKKICNLQRPEDEKLNIDLLTEFIRLTEHPSGSDLIYYPRDDREDSPEGIVKEIKEWRAANGKSGFKQGLEHHHHHH | MESKRNKPGKATGKGKPVGDRWLDDAGKDSGAPIPDRIADKLRDKEFKNFDDFRKKFWKEVAKDPDLAKQFKGGNRKSIKQGRAPFAPQKDQVGGRRTFELHHDKPISQDGGVYDMNNIRVTTPKRHIDIHRGK | 30 |
| des45 | 10 | 0.71 | 1507 | 0.71 | -40.2 | - | MELKHSISDYTEAEFLEFVKKIEENPENAELVEEFIRLTEHPSGSDLVYYPRDDREDSPEGIVKEIKEWRAANGKSGFKQGLEHHHHHH | MESKRNKPGKATGKGKPVGDKWLDDAGKDSGAPIPDRIADKLRDKEFKNFDDFRKKFWEEVSKDPDLAKQFNRQNQKNIKRGYAPFAREKDQVGGRRTFELHHDKPISQDGGVYDMNNIRVTTPKRHIDIHRGK | 29 |
| des46 | 15 | 0.70 | 1588 | 0.73 | -36.1 | - | MELKHSISDYTEAEFLEFVKKICELSGSESDTNNKLVEEFIRLTEHPSGSDLVYYPRDDREDSPEGIVKEIKEWRAANGKSGFKQGLEHHHHHH | MESKRNKPGKATGKGKPVGDRWLDDAGKDSGAPIPDRIADKLRDKEFKNFDDFRRKFWEEVSKDPDLAKQFKKGNRESIKRGYAPFAPQKDQVGGRKVFELHHDKPISQDGGVYDMNNIRVTTPKRHIDIHRGK | 28 |
| des47 | 12 | 0.67 | 1635 | 0.72 | -36.2 | - | MELKHSISDYTEAEFLEFVKKILTADGDTAEQLVSEFVRLTEHPSGSDLIYYPRDDREDSPEGIVKEIKEWRAANGKSGFKQGLEHHHHHH | MESKRNKPGKATGKGKPVGDKWLDDAGKDSGAPIPDRIADKLRDKEFKNFDDFRKKFWEEVSKDPDLAKQFNPQNRERIKRGQSPFAREKDQVGGRRRFELHHDKPISQDGGVYDMNNIRVTTPKRHIDIHRGK | 25 |
| des48 | 14 | 0.72 | 1541 | 0.69 | -35.8 | - | MELKHSISDYTEAEFLEFVKEIIDYSPTEWAREKLVTEFIRLTEHPSGSDLVYYPRDDREDSPEGIVKEIKEWRAANGKSGFKQGLEHHHHHH | MESKRNKPGKATGKGKPVGDRWLDDAGKDSGAPIPDRIADKLRDKEFKNFDDFRKKFWEEVSKDPDLAKQFKGSNRRNIKQGYAPFAPQKDQVGGRKTFELHHDKPISQDGGVYDMNNIRVTTPKRHIDIHRGK | 26 |
| des49 | 20 | 0.69 | 1758 | 0.71 | -41.7 | - | MELKHSISDYTEAEFLEFVKKIIDITEYGSPDQQEKQLPKLLTEFERLTEHPSGSDLIYYPRDDREDSPEGIVKEIKEWRAANGKSGFKQGLEHHHHHH | MESKRNKPGKATGKGKPVGDKWLDDAGKDSGAPIPDRIADKLRDKEFKNFDDFRKKFWKEVAKDPDLAKQFKPQNQASIKQGYAPFARQNDQVGGRRVFELHHDKPISQDGGVYDMNNIRVTTPKRHIDIHRGK | 34 |
| des50 | 12 | 0.68 | 1708 | 0.71 | -38.5 | - | MELKHSISDYTEAEFLEFVKKIEDSDPPDNRKLLEEFERLTEHPDGSDLIYYPRDDREDSPEGIVKEIKEWRAANGKSGFKQGLEHHHHHH | MESKRNKPGKATGKGKPVGDRWLDDAGKDSGAPIPDRIADKLRDKEFKNFDDFRKKFWEEVSKDPDLAKQFKGSNRKRIKQGQAPFARKNDQVGGRRTFELHHDKPISQDGGVYDMNNIRVTTPKRHIDIHRGK | 23 |
| des51 | 16 | 0.68 | 1605 | 0.73 | -38.3 | - | MELKHSISDYTEAEFLEFVKKIRELAKNGKTDESAELVREFKRLTEHPSGSDLVYYPRDDREDSPEGIVKEIKEWRAANGKSGFKQGLEHHHHHH | MESKRNKPGKATGKGKPVGDRWLDDAGKDSGAPIPDRIADKLRDKEFKNFDDFRKKFWKEVSKDPDLAKQFKGANRDNIKQGQSPFAPESQQVGGREKFELHHDKPISQDGGVYDMNNIRVTTPKRHIDIHRGK | 31 |
| des52 | 14 | 0.72 | 1680 | 0.69 | -36.7 | - | MELKHSISDYTEAEFLEFVKKIFRIKENPEEQERLVEEFERLTEHPSGSDLIYYPRDDREDSPEGIVKEIKEWRAANGKSGFKQGLEHHHHHH | MESKRNKPGKATGKGKPVGDKWLDDAGKDSGAPIPDRIADKLRDKEFKNFDDFRKKFWEEVSKDPDLSKQFKRSNQDNIKQGLAPFAPKKDQVGGRKRFELHHDKPISQDGGVYDMNNIRVTTPKRHIDIHRGK | 20 |
| des53 | 9 | 0.70 | 1529 | 0.76 | -39.7 | - | MELKHSISDYTEAEFLEFVKKIFNTGDEDLVEEFKRLTEHPSGSDLVYYPRDDREDSPEGIVKEIKEWRAANGKSGFKQGLEHHHHHH | MESKRNKPGKATGKGKPVGDRWLDDAGKDSGAPIPDRIADKLRDKEFKNFDDFRKKFWEEVSKDPDLAKQFKRANRDRIKQGQAPFAPEKDQVGGRKRFELHHDKPISQDGGVYDMNNIRVTTPKRHIDIHRGK | 29 |
| des54 | 16 | 0.71 | 1509 | 0.70 | -33.0 | - | MELKHSISDYTEAEFLEFVKKIREAAENGDYDLAHELVNKFEKLTEHPDGSDLVYYPRDDREDSPEGIVKEIKEWRAANGKSGFKQGLEHHHHHH | MESKRNKPGKATGKGKPVGDKWLDDAGKDSGAPIPDRIADKLRDKEFKNFDDFRKKFWEEVSKDPDLSKQFNPNNKKQIQKGYAPFAPNKDQVGGRRVFELHHDKPISQDGGVYDMNNIRVTTPKRHIDIHRGK | 26 |
| des55 | 14 | 0.69 | 1558 | 0.73 | -36.2 | - | MELKHSISDYTEAEFLEFVKEICDEGAPPSKEEKYVDEFKRLTEHPDGSDLIYYPRDDREDSPEGIVKEIKEWRAANGKSGFKQGLEHHHHHH | MESKRNKPGKATGKGKPVGDKWLDDAGKDSGAPIPDRIADKLRDKEFKNFDDFRKKFWEEVAKDPDLAKQFNPQNQKNIKRGLAPFARESDQVGGRNRFELHHDKPISQDGGVYDMNNIRVTTPKRHIDIHRGK | 25 |
| des56 | 17 | 0.71 | 1446 | 0.70 | -35.4 | - | MELKHSISDYTEAEFLEFVKKICKSVENGSPPDDQSKQVEEFKRLTEHPDGSDLIYYPRSDREDSPEGIVKEIKEWRAANGKSGFKQGLEHHHHHH | MESKRNKPGKATGKGKPVGDKWLDDAGKDSGAPIPDRIADKLRDKEFKNFDDFRKAFWKEVAKDPDLAKQFSKSNQGNIKNGRAPFAPESDQVGGRTTYELHHDKPISQDGGVYDMNNIRVTTPKRHIDIHRGK | 32 |
| des57 | 20 | 0.69 | 1672 | 0.72 | -41.5 | - | MELKHSISDYTEAEFLEFVKKIEEAIIKNPSKHSDGEMQQLVQEFERLTEHPDGSDLIFYPRSDREDSPEGIVKEIKEWRAANGKSGFKQGLEHHHHHH | MESKRNKPGKATGKGKPVGDKWLDDAGKDSGAPIPDRIADKLRDKEFKNFDDFRKKFWEEVAKDPDLAKQFNPRNQELIKAGRAPFARTSDQVGGRKTFELHHDKPISQDGGVYDMNNIRVTTPKRHIDIHRGK | 35 |
| des58 | 13 | 0.72 | 1582 | 0.70 | -32.5 | - | MELKHSISDYTEAEFLEFVKKITSQGQLKQNRPLVREFERLTEHPDGSDLIYYPRSDREDSPEGIVKEIKEWRAANGKSGFKQGLEHHHHHH | MESKRNKPGKATGKGKPVGDKWLDDAGKDSGAPIPDRIADKLRDKEFKNFDDFRKKFWLEVAKDPDLAKQFSSGNQANIKQGKAPFARKSDQVGGRETFELHHDKPISQDGGVYDMNNIRVTTPKRHIDIHRGK | 25 |
| des59 | 20 | 0.69 | 1826 | 0.74 | -39.9 | - | MELKHSISDYTEAEFLEFVKKIMKLINDQVNARRYTLLSKLVTEFERLTEHPDGSDLIYYPRDDREDSPEGIVKEIKEWRAANGKSGFKQGLEHHHHHH | MESKRNKPGKATGKGKPVGDKWLDDAGKDSGAPIPDRIADKLRDKEFKNFDHFREAFWKEVAKDPDLAKQFRPQNQALIKAGFAPFARKSDQVGGRRTFELHHDKPISQDGGVYDMNNIRVTTPKRHIDIHRGK | 36 |
