# Supplementary material for: Ultrahigh specificity in a network of computationally designed protein-interaction pairs
Source: Nat Commun. 2018 Dec 11;9:5286. doi: 10.1038/s41467-018-07722-9 (PMC6290019; doi:10.1038/s41467-018-07722-9)
Supplement: Supplementary file 5 — Reporting Summary [file 41467_2018_7722_MOESM5_ESM.pdf]

nature research

Corresponding author(s): *Double-blind peer review submissions: write DBPR and your manuscript number here instead of author names.*

Reporting Summary

Nature Research wishes to improve the reproducibility of the work that we publish. This form provides structure for consistency and transparency in reporting. For further information on Nature Research policies, see Authors & Referees and the Editorial Policy Checklist.

Statistical parameters

When statistical analyses are reported, confirm that the following items are present in the relevant location (e.g. figure legend, table legend, main text, or Methods section).

n/a	Confirmed	
☐	☒	The exact sample size (*n*) for each experimental group/condition, given as a discrete number and unit of measurement
☐	☒	An indication of whether measurements were taken from distinct samples or whether the same sample was measured repeatedly
☒	☐	The statistical test(s) used AND whether they are one- or two-sided<br>*Only common tests should be described solely by name; describe more complex techniques in the Methods section.*
☒	☐	A description of all covariates tested
☐	☒	A description of any assumptions or corrections, such as tests of normality and adjustment for multiple comparisons
☐	☒	A full description of the statistics including central tendency (e.g. means) or other basic estimates (e.g. regression coefficient) AND variation (e.g. standard deviation) or associated estimates of uncertainty (e.g. confidence intervals)
☒	☐	For null hypothesis testing, the test statistic (e.g. *F*, *t*, *r*) with confidence intervals, effect sizes, degrees of freedom and *P* value noted<br>*Give P values as exact values whenever suitable.*
☒	☐	For Bayesian analysis, information on the choice of priors and Markov chain Monte Carlo settings
☒	☐	For hierarchical and complex designs, identification of the appropriate level for tests and full reporting of outcomes
☒	☐	Estimates of effect sizes (e.g. Cohen's *d*, Pearson's *r*), indicating how they were calculated
☒	☐	Clearly defined error bars<br>*State explicitly what error bars represent (e.g. SD, SE, CI)*

Our web collection on statistics for biologists may be useful.

Software and code

Policy information about availability of computer code

Data collection	Rosetta design software was used for generating the proteins' models. BLASTP was used to construct the multiple sequence alignments and PSI-BLAST for generating the PSSMs. Biacore T200 evaluation software 3.0 was used for collecting and the SPR data. Crystallographic diffraction images were indexed and integrated using the Mosflm program, and scaled using the SCALA program. The structures were solved with the program PHASER80. All steps of atomic refinement were carried out with the CCP4/ REFMAC5 program and by Phenix refine. The models were built into maps using the COOT program.
Data analysis	Biacore T200 evaluation software 3.0 was used for analyzing and the SPR data and Prism 7 was used for making the plots. Crystallographic diffraction images were indexed and integrated using the Mosflm program, and scaled using the SCALA program. The structures were solved with the program PHASER80. All steps of atomic refinement were carried out with the CCP4/ REFMAC5 program and by Phenix refine. The models were built into maps using the COOT program.

For manuscripts utilizing custom algorithms or software that are central to the research but not yet described in published literature, software must be made available to editors/reviewers upon request. We strongly encourage code deposition in a community repository (e.g. GitHub). See the Nature Research guidelines for submitting code & software for further information.

Data

Policy information about availability of data

All manuscripts must include a data availability statement. This statement should provide the following information, where applicable:

- Accession codes, unique identifiers, or web links for publicly available datasets
- A list of figures that have associated raw data
- A description of any restrictions on data availability

Code availability
Rosetta is available free of charge to all academic users (http://www.rosettacommons.org). Rosetta git version 627f7dd22223c3074594934b789abb4f4e2e3b10 was used for all design simulations. All Rosetta modeling and design was done using RosettaScripts66 that are available with their command lines and flag files in Supplementary Data 2.

Data availability
The amino acid sequences and the computed Rosetta scores of the 59 designs that were tested experimentally and the wild type are available in Supplementary Data 1. The coordinates of the designs colEdes3/Imdes3 and colEdes7/Imdes7 are available from the RCSB Protein Data Bank with accession codes 6ERE [http://www.rcsb.org/pdb/results/results.do?tabtoshow=Unreleased&qrid=920943E3] and 6ER6 [http://www.rcsb.org/pdb/results/results.do?tabtoshow=Unreleased&qrid=35EE039B], respectively. Plasmids encoding the 18 successful designs and designed pair 3.5 were deposited in the AddGene repository (http://www.addgene.org).

Field-specific reporting

Please select the best fit for your research. If you are not sure, read the appropriate sections before making your selection.

☒ Life sciences ☐ Behavioural & social sciences ☐ Ecological, evolutionary & environmental sciences

For a reference copy of the document with all sections, see nature.com/authors/policies/ReportingSummary-flat.pdf

Life sciences study design

All studies must disclose on these points even when the disclosure is negative.

Sample size	We chose 59 computational designs for experiments since they provided a large enough set to test the algorithm performance and at the same time were within the reach of our in vivo viability screen. We chose 9 protein pairs for all-against-all SPR binding measurements since this assay is highly time consuming so that 81 interactions seemed like a challenging but possible higher boundary.
Data exclusions	For the majority of the interactions, SPR measurements were performed in 12 different analyte concentrations since the affinities were highly variable and we could not know in advance the dissociation constant of each interaction. We therefore excluded from the analysis concentrations that were too high or too low to allow correct determination of affinity.
Replication	For each SPR measurement, we measured a chosen analyte concentration at the beginning, middle and end of each run to verify chip stability and measurement reproducibility. In addition, we repeated the SPR measurements for many of the interactions, including preparation of new SPR chips, in order to verify the affinity values.
Randomization	This is irrelevant for our paper, which focuses on computational design and experimental testing of protein interaction networks.
Blinding	For the crystallographic structure determination of the designs, the researcher and software were blinded to the computational models.

Reporting for specific materials, systems and methods

Materials & experimental systems

n/a	Involved in the study
☐	☒ Unique biological materials
☒	☐ Antibodies
☒	☐ Eukaryotic cell lines
☒	☐ Palaeontology
☒	☐ Animals and other organisms
☒	☐ Human research participants

Methods

n/a	Involved in the study
☒	☐ ChIP-seq
☒	☐ Flow cytometry
☒	☐ MRI-based neuroimaging

Unique biological materials

Policy information about availability of materials

Obtaining unique materials	The amino acid sequences of the 59 designs that were tested experimentally and the wild type are available in Supplemental Data 1. Plasmids encoding the 18 successful designs and designed pair 3.5 were deposited in the AddGene repository (http://www.addgene.org)
